# Hsp90 Orchestrates Transcriptional Regulation by Hsf1 and Cell Wall Remodelling by MAPK Signalling during Thermal Adaptation in a Pathogenic Yeast

**DOI:** 10.1371/journal.ppat.1003069

**Published:** 2012-12-27

**Authors:** Michelle D. Leach, Susan Budge, Louise Walker, Carol Munro, Leah E. Cowen, Alistair J. P. Brown

**Affiliations:** 1 Aberdeen Fungal Group, School of Medical Sciences, University of Aberdeen, Institute of Medical Sciences, Foresterhill, Aberdeen, United Kingdom; 2 Department of Molecular Genetics, University of Toronto, Medical Sciences Building, Toronto, Ontario, Canada; University of Melbourne, Australia

## Abstract

Thermal adaptation is essential in all organisms. In yeasts, the heat shock response is commanded by the heat shock transcription factor Hsf1. Here we have integrated unbiased genetic screens with directed molecular dissection to demonstrate that multiple signalling cascades contribute to thermal adaptation in the pathogenic yeast *Candida albicans*. We show that the molecular chaperone heat shock protein 90 (Hsp90) interacts with and down-regulates Hsf1 thereby modulating short term thermal adaptation. In the longer term, thermal adaptation depends on key MAP kinase signalling pathways that are associated with cell wall remodelling: the Hog1, Mkc1 and Cek1 pathways. We demonstrate that these pathways are differentially activated and display cross talk during heat shock. As a result ambient temperature significantly affects the resistance of *C. albicans* cells to cell wall stresses (Calcofluor White and Congo Red), but not osmotic stress (NaCl). We also show that the inactivation of MAP kinase signalling disrupts this cross talk between thermal and cell wall adaptation. Critically, Hsp90 coordinates this cross talk. Genetic and pharmacological inhibition of Hsp90 disrupts the Hsf1-Hsp90 regulatory circuit thereby disturbing *HSP* gene regulation and reducing the resistance of *C. albicans* to proteotoxic stresses. Hsp90 depletion also affects cell wall biogenesis by impairing the activation of its client proteins Mkc1 and Hog1, as well as Cek1, which we implicate as a new Hsp90 client in this study. Therefore Hsp90 modulates the short term Hsf1-mediated activation of the classic heat shock response, coordinating this response with long term thermal adaptation via Mkc1- Hog1- and Cek1-mediated cell wall remodelling.

## Author Summary

*Candida albicans* is one of the most persistent yeast pathogens known to man, causing frequent mucosal infections (thrush) in otherwise healthy individuals, and potentially fatal bloodstream infections in immunocompromised patients. *C. albicans* colonises warm-blooded animals and occupies thermally buffered niches. Yet during its evolution this pathogen has retained the classic heat shock response whilst other stress responses have diverged significantly. We have established that the essential, evolutionarily conserved molecular chaperone, Hsp90, coordinates thermal adaptation. Hsp90 interacts with and modulates the activity of the heat shock transcription factor, Hsf1, thereby controlling the expression of heat shock proteins required for the clearance of proteins damaged by proteotoxic stresses. In addition, Hsp90 modulates the activities of key MAP kinase signalling pathways that mediate cell wall remodelling and long term adaptation to heat shock. Loss of any of these factors results in a significant reduction in thermotolerance.

## Introduction

Microorganisms inhabit dynamic environments and are continually challenged with environmental stimuli and stresses. Microbial survival depends upon effective environmental response strategies that have been elaborated over evolutionary time. These cellular strategies have been intensively studied in various contemporary model organisms [1], [2], [3], [4]. The emergent paradigm is that cells react to environmental changes via a sense and respond logic: they continuously monitor their environment, and upon encountering a stimulus, mount a cellular response [5]. This is achieved through diverse signalling pathways that drive physiological adaptation to a myriad of environmental stresses that include temperature fluctuations, osmotic, oxidative and weak acid stresses, as well as nutrient limitation [6], [7].

Fungal pathogens have evolved robust stress responses that enable them to counteract the antimicrobial defences of their host, thereby promoting the colonisation of specific niches. The major fungal pathogen of humans, *Candida albicans*, is an opportunistic pathogen that has evolved as a relatively harmless commensal of the mucous membranes and digestive tracts of healthy individuals [8], [9]. *C. albicans* is a common cause of mucosal infections (thrush) and when antimicrobial defences become compromised this yeast can cause life-threatening systemic infections [8], [10]. Stress responses are critical for survival of *C. albicans* inside the human body, and genetic inactivation of these responses attenuates virulence of this pathogen [11], [12], [13]. However, the regulation of these stress signalling mechanisms has diverged significantly in *C. albicans* compared with other yeasts [14]. For example, unlike *Saccharomyces cerevisiae, Schizosaccharomyces pombe* or *Candida glabrata* [2], [15], [16], *C. albicans* does not activate a large core transcriptional response [3]. The core transcriptional responses of *S. cerevisiae, S. pombe* and *C. glabrata* involve the activation of common sets of stress genes by one particular stress that promote cross-protection to diverse stresses [2], [15], [16]. In *S. cerevisiae* and *C. glabrata*, this core transcriptional response and hence stress cross-protection are dependent on the transcription factors Msn2 and Msn4, which activate target genes via stress response elements (STRE) in their promoters [17]. In *S. pombe*, the core stress response is driven largely by the Sty1 stress activated protein kinase (SAPK: the orthologue of Hog1 in other yeasts) [15]. In contrast, *C. albicans* does not mount a broad core transcriptional response to stress, there is limited stress cross-protection in this yeast, and the roles of Hog1 and Msn2/Msn4-like transcription factors have diverged in this pathogen [2], [3], [18], [19], [20]. Whilst, *C. albicans* does appear to activate a relatively specialised core transcriptional response to osmotic, oxidative and heavy metal stresses [19], the consensus view is that this pathogen mounts relatively specific responses to particular environmental challenges. This involves activation of the corresponding signal transduction pathway, subsequent activation of the relevant set of stress genes and requisite changes in cell physiology, morphology and adherence [21], [22], [23].

A number of stress regulatory modules have been conserved between *C. albicans* and other yeasts. For example, the AP1-like transcription factors *S. cerevisiae* Yap1 [24], *S. pombe* Pap1 [25], *C. glabrata* CgYap1 [26], [27] and *C. albicans* Cap1 [28] play analogous roles in the activation of transcriptional responses to oxidative stress. Also, the Hog1/Sty1 SAPK is conserved in these yeasts, although the orthologues have diverged with respect to the stress responses they regulate. *S. cerevisiae* Hog1 is primarily involved in responses to osmotic stress, whereas *C. albicans* Hog1 and *S. pombe* Sty1 contribute to a diverse range of stress responses [29], [30], [31]. Additional mitogen activated protein kinase (MAPK) cascades have been conserved between *S. cerevisiae* and *C. albicans.* These include the cell wall integrity Mpk1/Slt2 pathway [32] and the cell wall, morphogenesis and pheromone signalling pathways involving the Cek1 and Cek2 MAPKs [33], [34]. These MAP kinase pathways contribute to thermotolerance in *S. cerevisiae* [35], [36], [37], although the mechanisms by which they do so remain obscure. These MAP kinase pathways are also important for virulence, as MAP kinase defective *C. albicans* mutants display attenuated virulence in infection models [11], [13], [33], [38].

The heat shock response is among the most fundamentally important and ubiquitous stress responses in nature. The heat shock transcription factor (Hsf1) which drives this response, is conserved from yeasts to humans [39], [40]. Indeed, the Hsf1 module and the heat shock response are even conserved in *C. albicans,* an organism that is obligately associated with warm blooded mammals and hence occupies thermally buffered niches [41]. Furthermore Hsf1 is essential for viability in *C. albicans* [42] and other yeasts [43]. These observations reflect the fundamental importance of heat shock adaptation in all organisms. Even in the absence of stress, Hsf1 binds as a trimer to canonical heat shock elements (HSEs) in the promoters of target heat shock protein (*HSP*) genes [44], [45], [46]. When *S. cerevisiae* or *C. albicans* cells are exposed to an acute heat shock, Hsf1 becomes hyper-phosphorylated and activated, leading to the transcriptional induction of these target *HSP* genes, thereby promoting cellular adaptation to the thermal insult [39], [47].

Many HSPs are molecular chaperones that promote the folding, assembly, or cellular localisation of client proteins [48]. They also minimise the aggregation of unfolded or damaged proteins and often target such proteins for degradation [48]. HSPs are critical for the survival of eukaryotic cells under normal conditions as well as following exposure to an acute heat shock. Indeed our recent exploration of the dynamic regulation of Hsf1 during thermal adaptation has suggested that the Hsf1-HSE regulon is activated even during slow thermal transitions such as the increases in temperature suffered by febrile patients [49]. This explains why the Hsf1-HSE regulon is active in *C. albicans* cells infecting the mammalian kidney, and why activation of this regulon is essential for virulence of *C. albicans* [42]. Clearly the Hsf1-HSE regulon is critical for the maintenance of thermal homeostasis, not merely for adaptation to acute heat shocks.

Hsp90 has been suggested to play a critical role in regulation of the Hsf1-HSE regulon, contributing to an autoregulatory circuit involving Hsp90 and Hsf1 [49]. In the absence of stress, Hsp90 is generally expressed at relatively high levels [50], and is thought to repress Hsf1 [49]. However, thermal and other proteotoxic stresses can induce global problems in protein folding that overwhelm the functional capacity of Hsp90 [50]. Under these conditions the repression of Hsf1 by Hsp90 was proposed to be released, allowing Hsf1 to become activated leading to increased *HSP90* expression [49].

The repression of Hsf1 by Hsp90 was suggested by the observation that pharmacological inhibition of Hsp90 correlates with HSF1 activation in mammalian cells [51], [52]. Zou and colleagues demonstrated that HSF1 can be cross-linked to Hsp90 in unstressed HeLa cells, suggesting that HSF1 might interact directly with Hsp90 [52]. Additionally, the trimeric form of human HSF1 has been shown to associate with an Hsp90-immunophilin-p23 complex, and this is thought to repress HSF1 transcriptional activity [53]. Furthermore, HSP90 modulates HSF1 regulation in *Xenopus* oocytes [54]. Hence HSF1 is known to be a client protein of Hsp90 in metazoan cells. However, although mutations that interfere with Hsp90 function have been shown to derepress the expression of Hsf1-dependent reporter genes in *S. cerevisiae* [55], no physical interaction between Hsf1 and Hsp90 has been demonstrated in the fungal kingdom.

In this study, we explore the regulatory control of cellular circuitry governing the response to temperature stress through an integrated approach involving specific hypotheses and unbiased screens in *C. albicans*. We determined that Hsf1 is a client protein of Hsp90, establishing for the first time in the fungal kingdom that the Hsf1-Hsp90 interaction is critical for the regulation of short term adaptive responses to heat shock. We questioned how cells adapt to heat stress in the longer term by investigating which other signalling pathways contribute to thermotolerance and executing genetic screens for protein kinase mutations that confer temperature sensitivity. This revealed that the Hog1, Mkc1 and Cek1 MAP kinase pathways contribute to thermotolerance, as does casein kinase signalling. We show that these MAP kinase pathways are not essential for Hsf1 activation. Rather, they contribute to thermal adaptation in the longer term via cell wall remodelling. Hog1, Mkc1 and Cek1 are client proteins of Hsp90, and genetic depletion of Hsp90 affects this cell wall remodelling. Therefore, Hsp90 integrates the short term and long term molecular responses that underpin thermotolerance.

## Materials and Methods

### Strains and growth conditions

All strains are listed in Table 1, with the exception of the library of *C. albicans* transposon mutants [56], [57], [58]. Strains were grown in YPD (1% yeast extract, 2% bactopeptone, 2% glucose) [59]. To impose an instant heat shock of 30°C–42°C, cells were grown in YPD at 30°C to exponential phase, and mixed with an equal volume of medium that has been pre-warmed at 54°C in flasks which had been pre-warmed at 42°C. Cells were grown at 42°C for the times indicated. Doxycycline was added to YPD medium at a concentration of 20 µg/ml. Geldanamycin was added at 10 µM (A.G. Scientific, Inc., San Diego, USA), and radicicol at 20 µM (A.G. Scientific, Inc.).

**Table 1:** *C. albicans* strains.

Strain	Genotype	*Source*
ML250	*ade2::hisG/ade2::hisG, ura3::*λ*imm434/ura3::*λ*imm434, ENO1/eno1::ENO1-tetR-ScHAP4AD-3XHA-ADE2, HSF1/HSF1, pACT1-FLAG-HSF1*	[49]
SN95	*arg4Δ/arg4Δ his1Δ/his1Δ URA3/ura3::* λ*imm434 IRO1/iro1::*λ*imm434*	[102]
CaLC1411 (CaLC436)	*arg4Δ/arg4Δ his1Δ/his1Δ URA3/ura3::* λ*imm434 IRO1/iro1::* λ*imm434 HIS1/his1::tetR-FRT FRT-tetO-HSP90/hsp90::CdHIS1*	[73]
CaLC501	*arg4Δ/arg4Δ his1Δ/his1Δ URA3/ura3::* λ*imm434 IRO1/iro1::* λ*imm434 HSP90/HSP90-TAP-FRT HSF1/HSF1*	[73]
CaLC1819	*arg4Δ/arg4Δ his1Δ/his1Δ URA3/ura3::* λ*imm434 IRO1/iro1::* λ*imm434 HSP90/HSP90 ACT1p-3xFLAG-HSF1/HSF1*	this study
CaLC1875	*arg4Δ/arg4Δ his1Δ/his1Δ URA3/ura3::* λ*imm434 IRO1/iro1::*λ*imm434 HSP90/HSP90-TAP-FRT ACT1p-3xFLAG-HSF1/HSF1*	this study
CaLC1855	*arg4/arg4 his1/his1 URA3/ura3::imm434 IRO1/iro1::imm434 HSP90/HSP90-*GFP-NAT	this study
BWP17	*ura3::*λ*imm434/ura3::*λ*imm434, his1::hisG/his1::hisG, arg4::hisG/arg4::hisG*	[103]
CLM60-1	*ade2::hisG/ade2::hisG, ura3::*λ*imm434/ura3::*λ*imm434, ENO1/eno1::ENO1-tetR-ScHAP4AD-3XHA-ADE2, hsf1*::*hisG-URA3-hisG/HSF1*	[41]
*mkc1*Δ (CM1613C)	*mkc1*Δ::*hisG*-Ca*URA3-hisG/mkc1*Δ::*hisG ura3*Δ::λ*imm434/ura3*Δ::λ*imm434*	[32]
CaLC648	As SN95, *MKC1/MKC1-6xHIS-FLAG-FRT, HIS1/his1::TAR-FRT hsp90::CdHIS1/tetO-HSP90*	[86]
CaLC681	As SN95, *MKC1/MKC1-6xHIS-FLAG-FRT*	[86]
*hog1*Δ (JC50)	*ura3::*λ*imm434/ura3::*λ*imm434, his1::hisG/his1::hisG, hog1::loxP-ura3-loxP/hog1::loxP-HIS1-loxP* CIp20	[30]
*cek1*Δ (CK43B16)	*ura3/ura3 cek1*Δ*::hisG-URA3-hisG/cek1*Δ*::hisG*	[33]
*cst20*Δ (CDH22)	*ura3/ura3 cst20*Δ*::hisG-URA3-hisG/cst20*Δ*::hisG*	[104]
*cka1*Δ (VIC108)	*cka1Δ::ARG4*/*cka1Δ::URA3*	[61]
*cka2*Δ (VIC84)	*cka2Δ::ARG4/cka2Δ::URA3*	[61]
*ckb1*Δ (VIC138)	*ckb1Δ::ARG4/ckb1Δ::URA3*	[61]
*ckb2*Δ (VIC150)	*ckb2Δ::ARG4/ckb2Δ::URA3*	[61]
CAI4	*ura3::*λ*imm434/ura3::*λ*imm434*	[105]
CAI4+CIp10 (NGY152)	*ura3::λimm434/ura3::*λ*imm434 (RPS10-CIp10-URA3)*	[106]
MLC67	*ura3Δ::λimm434/ura3Δ::λimm434 RPS1::*p*ACT1-3xFLAG-HSF1/HSF1*	This study
MLC15	*ura3::* λ*imm434/ura3::*λ*imm434, his1::hisG/his1::hisG, hog1::loxP-ura3-loxP/hog1::loxP-HIS1-loxP,* RPS1::pACT1-FLAG-*HSF1*(NAT1)	This study
MLC21	*ura3/ura3 cek1*Δ*::hisG-URA3-hisG/cek1*Δ*::hisG,* RPS1::pACT1-FLAG-*HSF1*(NAT1)	This study
MLC24	*cka1*Δ*::ARG4*/*cka1Δ::URA3,* RPS1::pACT1-FLAG-*HSF1*(NAT1)	This study
MLC27	*cka2*Δ*::ARG4/cka2Δ::URA3,* RPS1::pACT1-FLAG-*HSF1*(NAT1)	This study
MLC30	*mkc1*Δ*:: hisG/mkc1Δ::hisG ura3*Δ*::λimm434/ura3*Δ*::λimm434,* RPS1::pACT1-FLAG-*HSF1*(NAT1)	This study
CaLC2259	*ckb21::ARG4/ckb21::URA3*, RPS1::pACT1-FLAG-*HSF1*(NAT1)	This study
CaLC2261	*ckb22::ARG4/ckb22::URA3*, RPS1::pACT1-FLAG-*HSF1*(NAT1)	This study
*hog1/hst7*Δ (CHH13)	*ura3*Δ::*imm434*/*ura3*Δ::*imm434 hst7*Δ::*hisG*/*hst7*Δ::*hisG hog1*::*hisG*-*URA3*-*hisG*/*hog1*::*hisG*	[81]
*cap1*Δ (JC128)	*ura3::* λ*imm434/ura3::*λ*imm434, his1::hisG/his1::hisG, arg4::hisG/arg4::hisG, cap1::hisG/cap1::hisG-URA3-hisG*	[19]
CaLC2287	*arg4/arg4 his1/his1 URA3/ura3::imm434 IRO1/iro1::imm434, CEK1/CEK1-TAP-ARG4*	This study
CaLC2288	*arg4/arg4 his1/his1 URA3/ura3::λimm434 IRO1/iro1::λimm434, HIS1/his1::TAR-FRT, hsp90::CdHIS1/FRT-tetO-HSP90, CEK1/CEK1-TAP-ARG4*	This study

### Strain construction

For co-immunoprecipitation of Hsf1 and Hsp90, Hsf1 was tagged with FLAG and Hsp90 was tagged with the tandem affinity purification (TAP) tag. The plasmid pACT1pHSF1, containing the *ACT1p-3xFLAG-HSF1* construct [41] was linearized with StuI and transformed into the strains SN95 (wild type) or CaLC501 (*HSP90-TAP*) (Table 1) using published procedures [60]. Nourseothricin (NAT) resistant transformants were selected on YPD containing 150 µg/mL NAT, and insertion of the *ACT1p-3xFLAG-HSF1* cassette confirmed by diagnostic PCR using the primers oLC1117/oLC1628 (Table S1).

Localisation of Hsp90 was achieved by 3′-tagging of one *HSP90* allele with GFP in the wild type strain SN95 (creating CaLC1855, Table 1). The GFP-NAT cassette was amplified using primers oLC1616/1617 (Table S1) and transformed into SN95. Proper integration of the cassette in NAT resistant transformants was confirmed by PCR using primer pairs oLC600/756 (Table S1).

To determine Hsf1 phosphorylation status, the pACT1pHSF1, containing the *ACT1p-3xFLAG-HSF1* construct [41] was linearized with StuI and transformed into *mkc1*, *hog1*, *cek1*, *cka1*, *cka1*, *ckb1*, *ckb2* mutants (Table 1) [30], [32], [33], [61]. NAT resistant transformants were selected and confirmed by diagnostic PCR and expression of FLAG-Hsf1 in western blots.

Cek1 was tagged with the TAP tag at its C-terminus in the wild type strain SN95 (creating CaLC2287, Table 1) and its derivative CaLC1411 (creating CaLC2288, Table 1) using a PCR based strategy as described previously [62]. Briefly, the tag and a selectable marker (*ARG4*) were PCR amplified from pLC573 (pFA-TAP-*ARG4* [63]) using oligos oLC2292/2251 (Table S1). 200 µl of PCR product was run through a PCR clean-up and dissolved in 50 µl sterile water and transformed into *C. albicans*. Correct genomic integration was verified using appropriate primer pairs that anneal ∼500 bp up (oLC2252) or downstream (oLC2253) from both insertion junctions together with oLC1593 (TAP-R) and oLC1594 (*ARG4*-F) that target the TAP and the selectable marker (Table S1).

### Western blotting

Total soluble protein was extracted and subjected to western blotting using published protocols [64], [65]. Briefly, mid-log cells were pelleted by centrifugation, washed with sterile water, and resuspended in lysis buffer (50 mM HEPES, pH 7.5, 150 mM NaCl, 5 mM EDTA, 1% Triton X-100). An equal volume of 0.5-mm acid-washed beads was added to each tube. Cells were mechanically disrupted on a BioSpec (Bartlesville, OK) mini-bead-beater for six 30 second intervals, with 1 minute on ice between each cycle. The beads and cell debris were pelleted by high-speed centrifugation and the supernatant removed for analysis. Protein concentration was determined using a Bradford reagent (Sigma-Aldrich) assay. Protein samples were mixed with one-sixth volume of 6× sample buffer containing 0.35 M Tris-HCl, 10% (w/w) SDS, 36% glycerol, 5% β-mercaptoethanol, and 0.012% bromophenol blue. Between 2 µg and 30 µg of protein was loaded in wells of an 8% SDS–PAGE gel. Separated proteins were transferred to a PVDF membrane for 1 hour at 100 V at 4°C. Membranes were blocked in 5% milk or 5% bovine serum albumin (BSA) in TBS or PBS containing 0.1% Tween-20 at room temperature for 1 hour and subsequently incubated in primary antibody as follows. All primary antibodies (except those against p38 and p44/42 MAPK) were left on the membrane for one hour at room temperature. The p38 MAPK and p44/42 MAPK antibodies were incubated overnight at 4°C. Membranes were washed with 1×PBS-T or TBS-T and probed for one hour with secondary antibody dissolved in 1×PBS-T and 5% milk or BSA. Membranes were washed in PBS-T and signals detected using an ECL western blotting kit as per the manufacturer's instructions (Pierce).

To detect FLAG-Hsf1, a 1∶25000 dilution of anti-FLAG HRP conjugated antibody (Sigma, A8592) was used in PBS-T+5% milk [PBS 0.1% Tween-20, 5% (w/v) milk]. To detect Act1, an anti-Act1 antibody was used (Santa Cruz Biotechnology, sc47778) at a 1∶1000 dilution in PBS-T+5% milk. To detect Hsp90, a 1∶10000 dilution of anti-Hsp90 antibody was used (courtesy of Bryan Larson) in PBS-T+5% milk. To detect Hsp70, a 1∶1000 dilution of anti-Hsp70 antibody (Enzo Life Sciences, ADI-SPA-822) was used in PBS-T+5% milk.

To detect Mkc1 and Cek1 phosphorylation [22], [66], a 1∶2000 dilution of phospho-p44/42 MAPK (Erk1/2) (Thr202/Tyr204) Rabbit mAb was used (New England Biolabs, Hitchin, Hertfordshire, UK, #4370) in TBS-T+5% BSA [TBS 0.1% Tween-20, 5% (w/v) BSA]. For Hog1 phosphorylation [30], a 1∶2000 dilution of phospho-p38 MAPK (Thr180/Tyr182) rabbit mAB was used in TBS-T+5% BSA (New England Biolabs, Hitchin, Hertfordshire, UK, #9211).

To detect Mkc1-6XHis-FLAG, the anti-FLAG-HRP antibody was used as above. To detect total Hog1 an anti-Hog1 antibody (Santa Cruz Biotechnology, y-215) was diluted 1∶1000 in PBS-T+5% milk. TAP-tagged Cek1 was detected using a 1∶5000 dilution of anti-TAP tag rabbit polyclonal antibody (Thermoscientific, CAB1001) in PBS-T+5% milk.

### Co-immunoprecipitations

*C. albicans* cultures were grown to mid-log phase (OD_600_ = 0.5), cells harvested, washed with sterile H_2_0 and resuspended in 1 ml of lysis buffer (20 mM Tris pH 7.5, 100 mM KCl, 5 mM MgCl and 20% glycerol, with one protease inhibitor cocktail per 50 ml (complete, EDTA-free tablet, Roche Diagnostics, Indianapolis, IN, USA), 1 mM PMSF (EMD Chemicals, Gibbstown, NJ, USA) and 20 mM sodium molybdate (Sigma Aldrich Co., St Louis, MO, USA)). Cells were then disrupted by bead beating twice for 4 minutes with a 7 minute break on ice between cycles. Lysates were centrifuged at 1300×g for three 5-minute cycles, recovering the supernatants at each stage. The combined lysate was then cleared by centrifugation at 21,000×g for 10 minutes at 4°C and protein concentrations determined using the Bradford assay [67].

Anti-FLAG immunoprecipitations were performed by diluting protein samples to 2 mg/ml in lysis buffer containing 20 mM sodium molybdate and 0.2% Tween, and incubating with anti-FLAG M2 affinity agarose (Sigma Aldrich) at 4°C overnight as per the manufacturer's specifications. Unbound material was discarded, the beads washed five times with 1 ml lysis buffer containing 0.1% Tween, and the bound proteins eluted by boiling in one volume of 2× sample buffer (125 mM Tris-HCl, pH 6.8, 5% glycerol, 2.5% SDS, 2.5% beta-mecarptoethanol, dH_2_O, bromophenol blue). Anti-IgG immunoprecipitations were performed using the same approach, but using rabbit IgG agarose (Sigma Aldrich) as per the manufacturer's specifications.

Protein samples were then electrophoresed on 8% SDS-PAGE gels. Proteins were then electrotransferred to PVDF membranes (Bio-Rad Laboratories, Inc., Hercules, CA, USA) and blocked with PBS-T+5% milk. Blots were incubated with antibodies against CaHsp90 (courtesy of Bryan Larsen) (1∶10000 dilution, [68]), or FLAG (1∶10000, Sigma Aldrich Co.).

### qRT-PCR

To monitor gene expression changes in response to *tetO*-*HSP90* depletion, strains SN95 and CaLC1411 were grown overnight at 30°C in YPD while shaking at 200 rpm. Stationary phase cultures were split, adjusted to an OD_600_ of 0.04 where one culture was treated with doxycycline (BD Biosciences), while the other was left untreated. Cells were grown for 7 hours at 30°C. To monitor gene expression changes in response to heat shock, wild type and SN95 cells were grown to mid-log phase, subjected to a 30°C–42°C heat shock and 50 ml was harvested from each culture at the specified time, centrifuged at 3000 rpm for 2 minutes at 4°C, washed once with dH_2_O before being frozen at −80°C. RNA was then isolated using the QIAGEN RNeasy kit and cDNA synthesis was performed using the AffinityScript cDNA synthesis kit (Stratagene). PCR was carried out using the SYBR Green JumpStart Taq ReadyMix (Sigma-Aldrich) with the following cycle conditions: 94°C for 2 minutes, and 94°C for 15 seconds, 60°C for 1 minute, 72°C for 1 minute, for 40 cycles. All reactions were done in triplicate using the following primer pairs: *HSP104* (oLC1620/1621), *HSP90* (oLC754/755), *PGA13* (oLC2256/2257), *PMT4* (oLC2262/2263), *RHR2* (oLC2266/2267). Transcript levels were normalised to *ACT1* (oLC2285/2286) (Table S1). Data were analysed in the StepOne analysis software (Applied Biosystems).

### Temperature sensitivity screen

The *C. albicans* transposon insertion mutant library was generously provided by Aaron Mitchell (Carnegie Mellon University) [56], [58]. Strains were inoculated in 100 µl YPD in 96 well plates and incubated overnight at 30°C with shaking at 200 rpm. Cells were then diluted 1∶10 in YPD and incubated at 30°C with shaking at 200 rpm for 4 hours. After 4 hours of exponential growth, cells were then exposed to a one hour 30°C–42°C heat shock by addition of 100 µl pre-warmed YPD at 54°C and incubating at 42°C, and the growth of each culture monitored continuously for 6 hours by measuring the OD_600_. Non-heat shocked control cells received 100 µl YPD at 30°C and were incubated at 30°C. This screen was repeated independently three times, and only those mutants that displayed consistent phenotypes were taken for further analysis. The validity of key transposon mutants highlighted by this screen was then confirmed by subjecting corresponding homozygous null mutants to the same screen.

### Minimum inhibitory concentration assays and stress phenotypes

Unless otherwise stated, the susceptibilities of strains were determined with the following stressors: heat stress (42°C heat shock), Calcofluor White (CFW: 100 µg/ml), Congo Red (CR: 100 µg/ml), H_2_O_2_ (5 mM) and NaCl (1 M). All stress assays were performed in YPD.

MIC assays were performed in flat bottom, 96-well microtiter plates (Corning Costar). Briefly, assays were set up in 0.2 ml/well, with 2× concentrations of NaCl, CFW and CR prepared in 100 µl YPD. Final concentrations of NaCl were 0, 0.25, 0.5, 1.0, 1.5 and 2 M; and for CFW and CR were 0, 25, 50, 100, 150 and 200 µg/ml. Cell densities of overnight cultures were determined and dilutions were prepared in YPD such that ∼10^3^ cells were inoculated into each well. Plates were placed statically at either 25°C, 30°C, 37°C or 42°C for 48 hours, after which plates were sealed and cells resuspended by agitation. Absorbance was determined at 600 nm using a spectrophotometer (VERSA max, Molecular Devices), and was corrected for background from the corresponding medium. Every strain was tested in duplicate on three separate occasions. MIC data were quantitatively displayed with colour using the program Java TreeView 1.1.6 (http://jtreeview.sourceforge.net).

For stress cross-protection assays, cells were grown overnight in YPD at 30°C with shaking at 200 rpm. These were diluted to an OD_600_ = 0.2 in fresh YPD, and grown for a further 4 hours at 30°C. Cells were then subjected to a 30 minute heat shock at 42°C, incubated at 30°C and stressed for one hour with CFW, CR, H_2_O_2_ or NaCl. Cells were diluted, plated onto YPD and viability determined (CFUs). Control cells were not subjected to the prior heat shock. In other experiments cells were exposed to a prior CFW, CR, H_2_O_2_ or NaCl stress for one hour at 30°C before being heat shocked at 42°C for 30 min, and then cell viability determined.

To test stress sensitivity following Hsp90 depletion, *C. albicans tetO*-*HSP90* cells (CaLC1411: Table 1) were incubated for 7 hours in YPD containing 20 µg/ml doxycycline at a starting OD_600_ of 0.04. These cells were left untreated or stressed for one hour with CFW, CR, a 30°C–42°C heat shock, H_2_O_2_ or NaCl at the concentrations specified above, and CFUs determined. Controls included CaLC1411 cells that were not treated with doxycycline, and parental SN95 cells that were subjected to the same treatments. Growth curves of CaLC1411 (tet*O-HSP90/hsp90*) were determined in the absence and presence of doxycycline to ensure adequate viability of this strain (Supplementary Figure S1C).

To determine kinase activation in response to Hsp90 depletion, wild type SN95 and *tetO-HSP90* (CaLC1411) were grown overnight in YPD at 30°C. Cells were diluted in YPD with or without doxycycline at a starting OD_600_ of 0.04 and incubated for 7 hours. Cells were then left untreated or stressed with H_2_O_2_ for 10 minutes and NaCl for 12 minutes to determine Hog1 activation. For Mkc1 and Cek1 activation, tagged versions of these proteins in SN95 and *tetO-HSP90* (CaLC1411) were grown as above and stressed with CFW for 30 minutes or a 30°C–42°C heat shock for 30 minutes. Mkc1 total levels were assayed using Mkc1-6XHis-FLAG tagged in SN95 and *tetO-HSP90* (CaLC681 and CaLC648 respectively, Table 1). Total Cek1 levels were assayed using Cek1-TAP tagged in SN95 and *tetO-HSP90* (CaLC2287 and CaLC2288 respectively, Table 1).

### Chitin content

The chitin content of cells was measured as described previously [69]. Briefly, formalin fixed cells were stained with 25 µg/ml Calcofluor White and fluorescence was preserved with Vectashield mounting medium (Vector Laboratories, Peterborough, United Kingdom). All samples were examined by differential interference contrast (DIC) and fluorescence microscopy (456 nm) with a Zeiss Axioplan 2 microscope. Images were captured using a C4742-95 digital camera (Hamamatsu Photonics, Hamamatsu, Japan) and analysed using Openlab software (version 4.04: Improvision, Coventry, United Kingdom). CFW fluorescence was quantified for 50 individual yeast cells from each sample, using region-of-interest measurements. Mean fluorescence intensities were then calculated and expressed as arbitrary units.

### Fluorescence microscopy

Exponentially growing Hsp90-GFP (CaLC1855, Table 1) cells were heat shocked as described above, and 1 ml of cells were removed at 0, 10, 60 and 120 minutes post-heat shock. Cells were harvested and washed in 1 ml of 42°C pre-warmed 1×PBS. Supernatant was removed and cells were resuspended in the remaining 50 µl. Of this, 5 µl was placed on a slide, and cells were heat fixed by placing the slide on a 70°C hot plate for 1 minute. Slides were cooled for a few seconds before 4 µl of 1 µg/ml DAPI (Fluke, Sigma-Aldrich) were added. Imaging was performed on a Zeiss Imager M1 upright microscope and AxioCam MRm with AxioVision 4.7 software. An X-Cite series120 light source with ET green fluorescent protein (GFP) and 4′,6-diamidino-2-phenylindole (DAPI) hybrid filter sets from ChromaTechnology (Bellows Falls, VT) were used for fluorescence microscopy. DAPI fluorescence was viewed under the DAPI hybrid filter and GFP-tagged proteins under the GFP filter.

### Transmission electron microscopy

For high pressure freezing transmission electron microscopy (HPF-TEM), cells were prepared by high-pressure freezing with a Leica EM PACT2 (Leica Microsystems (UK) Ltd, Milton Keynes). After freezing, cells were freeze-substituted in substitution reagent (1% OsO4/0.1% uranyl acetate in acetone) with a Leica EM AFS2. Samples were encapsulated in 3% (w/v) low melting point agarose prior to processing to Spurr resin. Additional infiltration was provided under vacuum at 60°C before embedding in TAAB capsules and polymerizing at 60°C for 48 h. Semi-thin survey sections of 0.5 µM thickness were stained with 1% toluidine blue to identify areas of best cell density. Ultrathin sections (60 nm) were prepared with a Diatome diamond knife on a Leica UC6 ultramicrotome, and stained with uranyl acetate and lead citrate for examination with a Philips CM10 transmission microscope (FEI UK Ltd, Cambridge, UK) and imaging with a Gatan Bioscan 792 (Gatan UK, Abingdon, UK).

## Results

### Hsp90 negatively regulates Hsf1

**Figure 1:**
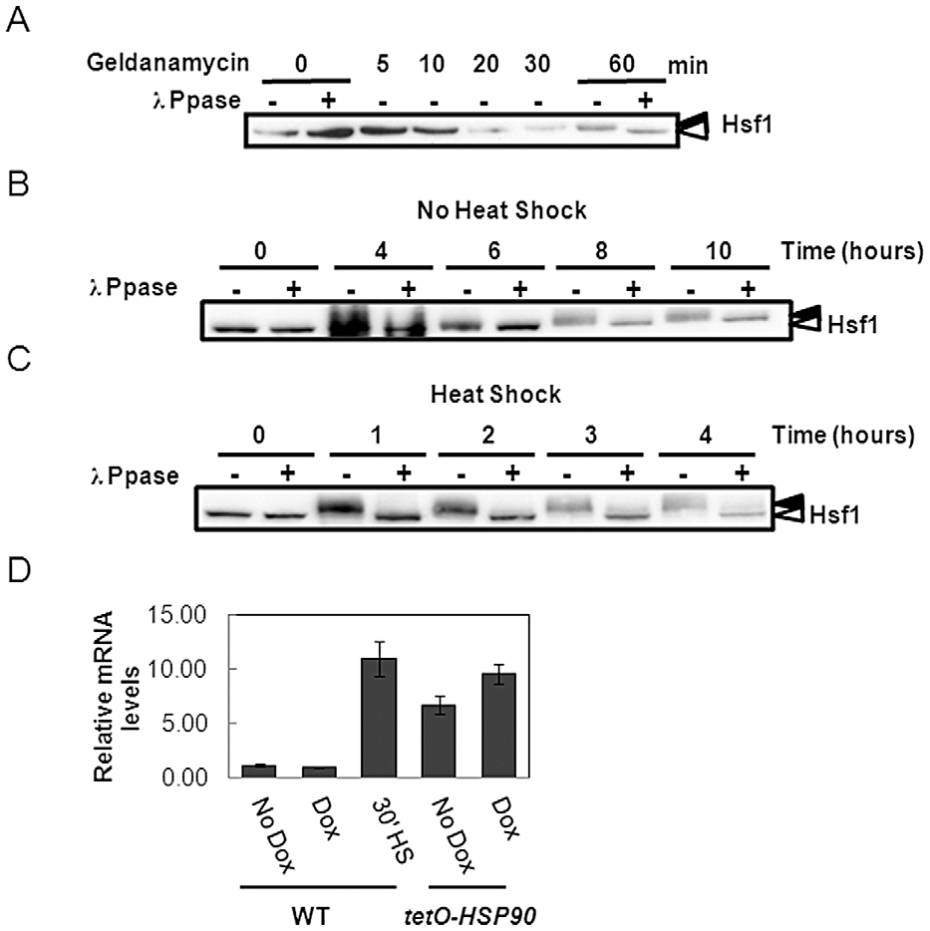
Hsf1 down-regulates Hsp90. (A) Inhibiting Hsp90 with geldanamycin leads to Hsf1 activation. *C. albicans* cells were incubated with 20 µM geldanamycin for one hour, and Hsf1 phosphorylation examined by western blotting. (B) Depleting Hsp90 leads to Hsf1 activation. Doxycycline treatment of *C. albicans tetO-HSP90* cells was used to ectopically down-regulate Hsp90 levels, and Hsf1 phosphorylation was then examined by western blotting: black arrow, phosphorylated Hsf1; white arrow, non-phosphorylated Hsf1. Hsf1 phosphorylation was confirmed using lambda phosphatase controls. (C) Hsp90 depletion causes protracted Hsf1 phosphorylation during a 30°C–42°C heat shock. Exponentially growing *C. albicans tetO-HSP90* cells were transferred from 30°C to 42°C, and 20 µg/ml doxycycline was added. Hsf1 phosphorylation was examined every hour up to 4 hours. Data reflect the outcomes for at least two independent replicate experiments. (D) *HSP104* transcript levels increase following Hsp90 depletion in *tetO-HSP90* cells. WT (wild type, SN95: Table 1) and *tetO-HSP90* cells (CaLC1411) were treated with 0 or 20 µg/ml doxycycline for 7 hours, and *HSP104* transcript levels were measured and normalised to the *ACT1* loading control. Wild-type cells were also subjected to a 30 minute 30°C–42°C heat shock for *HSP104* comparison.

An autoregulatory loop, whereby Hsf1 activates *HSP90* expression and Hsp90 interacts with and down-regulates Hsf1, is thought to lie at the heart of heat shock adaptation in fungi. This presumption provided the basis for mathematical modelling of thermal adaptation in *C. albicans* [49], but had not been confirmed experimentally. If this presumption is true, one would expect that inhibition of Hsp90, or Hsp90 depletion would lead to Hsf1 activation [49]. To test this, we examined the impact of the Hsp90 inhibitors, radicicol and geldanamycin [70], [71], upon Hsf1 phosphorylation. *C. albicans* cells (ML250: Table 1) were incubated with radicicol or geldanamycin for up to one hour, and Hsf1 phosphorylation was monitored via the resultant band shift revealed by western blot analysis, as described previously [41] (Figure 1A). These Hsp90 inhibitors induced Hsf1 phosphorylation after one hour, as confirmed by the band shift observed after controlled dephosphorylation with lambda phosphatase. To exclude the possibility that this Hsf1 phosphorylation was induced by a general effect of the drugs upon protein folding, we examined the impact of dithiothreitol and tunicamycin, known inducers of the unfolded protein response in *C. albicans* [72]. Hsf1 was not activated following treatment with 5 mM dithiothreitol or 4.73 µM tunicamycin for 1 hour (data not shown), suggesting that the induction of Hsf1 phosphorylation by radicicol and geldanamycin related to Hsp90 function rather than some general effect upon protein folding. Therefore, Hsp90 inhibits Hsf1, as predicted.

Next, we validated our pharmacological findings using a genetic approach. A doxycycline conditional *C. albicans HSP90* mutant (*tetO-HSP90*) [73], in which *HSP90* expression is independent of Hsf1, was used to ectopically down-regulate Hsp90 levels (Figure 1B and S1A). Doxycycline treatment led to Hsf1 phosphorylation after approximately 6 hours, at which point Hsp90 levels were reduced by 50% (Figure 1B and S1A). Therefore, ectopic down-regulation of *HSP90* caused Hsf1 phosphorylation even in the absence of a heat shock, further reinforcing the hypothesis that Hsp90 inhibits Hsf1.

A key finding from our modelling of thermal adaptation was that this system displays perfect adaptation: i.e. Hsf1 activation returns to basal levels within two hours once cells have adapted to their new ambient temperature [49]. If Hsp90 down-regulates the heat shock response, then one would expect this perfect adaptation to be dependent on Hsp90. Also, if new Hsp90 synthesis is inhibited after a heat shock, Hsf1 would remain phosphorylated and the system would not adapt to this stress. We tested this by examining Hsf1 phosphorylation levels in doxycycline-treated *C. albicans tetO-HSP90* cells after a 30°C–42°C heat shock. As predicted, these Hsp90-depleted cells were unable to recover, as revealed by the maintenance of Hsf1 phosphorylation four hours after the heat shock (Figure 1C). Therefore, perfect thermal adaptation is dependent upon Hsp90.

To further test the impact of depleting Hsp90 on the heat shock response, we looked at induction of *HSP104*, a known target of Hsf1 [49]. In wild type cells (SN95: Table 1) *HSP104* was up-regulated approximately 10-fold in response to a 30 minute 30°C–42°C heat shock (Figure 1D), which was consistent with previous findings [49], and *HSP104* expression was not affected by doxycycline treatment. *HSP104* mRNA levels were elevated in *tetO-HSP90* cells even in the absence of doxycycline, presumably because Hsp90 levels are significantly reduced under these conditions (Figure S1B). An additional increase in *HSP104* expression was observed after doxycycline treatment when Hsp90 levels were reduced further (Figure S1B). Taken together, these data strongly imply that Hsp90 is a master regulator of the heat shock response.

**Figure 2:**
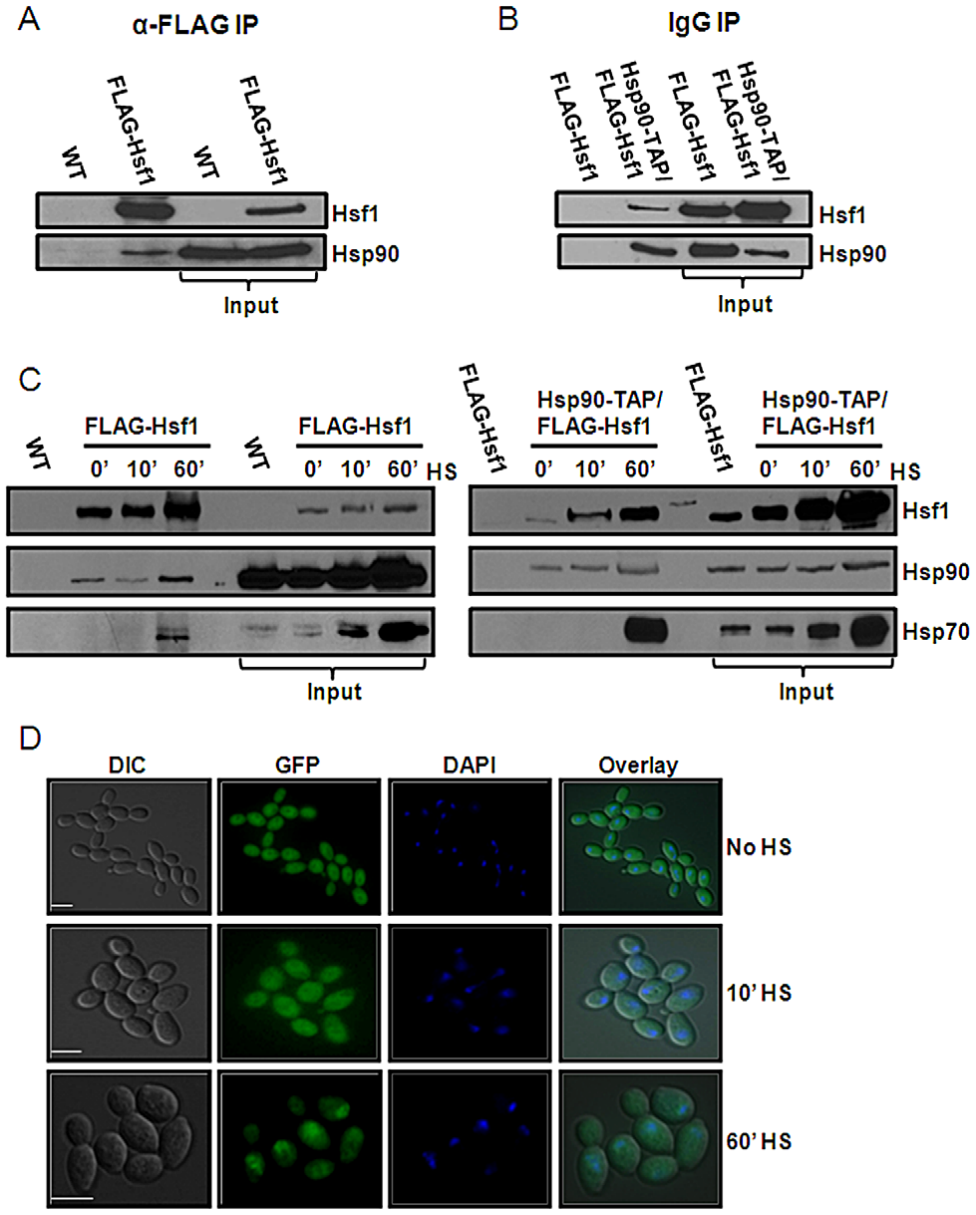
Hsf1 is an Hsp90 client in *C. albicans*. Hsf1 and Hsp90 physically interact as revealed by co-immunoprecipitation. (A) Hsp90 co-purifies with FLAG-Hsf1 after immunoprecipitation of extracts from unstressed cells with anti-FLAG M2 affinity agarose. Hsp90 was not co-immunoprecipitated from control cells lacking tagged Hsf1. (B) Reciprocal co-immunoprecipitation was performed using Hsp90-TAP showing that FLAG-Hsf1 copurifies with Hsp90-TAP upon immunoprecipitation with IgG agarose. FLAG-Hsf1 was not co-immunoprecipiated with IgG agarose in control cells lacking FLAG-Hsf1. Data reflect the outcomes for three independent replicate experiments. (C) Hsp90 co-immunoprecipitates with FLAG-Hsf1 0, 10 and 60 minutes after a 30°C to 42°C heat shock, but does not co-immunoprecipitate in control cells lacking FLAG-Hsf1. The membrane was re-probed for Hsp70, which co-purifies with FLAG-Hsf1 at 60 minutes post-heat shock. The reciprocal co-immunopreciptation validates these results: FLAG-Hsf1 co-immunoprecipitates with Hsp90-TAP at 0, 10 and 60 minutes post-heat shock. Re-probing the membranes for Hsp70, reveals that Hsp70 interacts with Hsp90-TAP at 60 minutes post-heat shock. (D) Localisation of Hsp90-GFP in response to elevated temperature. *C. albicans* CaLC1855 cells were treated with a 42°C heat shock and fixed at 0, 10 and 60 minutes post-heat shock. Hsp90 is localised in the cytosol at 0 and 10 minutes post-heat shock. Significant accumulation of Hsp90 in the nucleus is observed 60 minutes post-heat shock, as determined by co-staining with DAPI. Scale bars, 5 µm.

The proposed Hsf1-Hsp90 autoregulatory loop [49] suggests a physical interaction between Hsf1 and Hsp90. There is some evidence for this in mammalian cells [52], [53], but none in fungal systems. Therefore we tested this experimentally by co-immunoprecipitation. First, proteins were extracted from *C. albicans* cells expressing a FLAG-tagged Hsf1 protein (CaLC1819), and from control cells in which Hsf1 was not FLAG-tagged (WT, SN95). Protein extracts were incubated with anti-FLAG beads, and the resulting immunoprecipitates probed for Hsp90 on western blots. Hsp90 was observed reproducibly in immunoprecipitates from the FLAG-Hsf1 expressing strain, but not in those from the untagged control strain (Figure 2A). Reciprocal immunoprecipitations were then performed to test the validity of this apparent Hsf1-Hsp90 interaction. *C. albicans* cells expressing both TAP-tagged Hsp90 and FLAG-tagged Hsf1 (CaLC1875) were used in these experiments, and cells lacking the FLAG-tagged Hsf1 (CaLC501: Table 1) were used as a control. Hsp90 was immunoprecipitated using IgG beads, which bind the Protein A in the TAP tag. These immunoprecipitates were then probed for the FLAG-tagged Hsf1, revealing a band of the appropriate mass from cells expressing TAP-tagged Hsp90, but not from the controls (Figure 2B). This confirmed that Hsp90 interacts physically with Hsf1. This is the first demonstration of a physical interaction between Hsf1 and Hsp90 in any yeast.

An Hsf1-Hsp90 autoregulatory loop, as inferred by the model [49], [74], predicts dynamic changes in the Hsf1-Hsp90 interaction during a heat shock. The model predicts that Hsp90 releases Hsf1 following a heat shock, rebinding Hsf1 as the response is down-regulated. We tested this hypothesis by co-immunoprecipitation, examining Hsp90-Hsf1 interactions over a 60 minute period following heat shock. Rather than decreasing when Hsf1 becomes activated (as was predicted), the Hsp90-Hsf1 interaction increased over 60 minutes (Figure 2C) during the period when Hsf1 activation is maximal [49]. Therefore, Hsf1 is not released from Hsp90 after heat shock, and this mechanism cannot account for Hsf1 activation. We examined the Hsf1-Hsp90 interaction two hours after heat shock, when the response is down-regulated [49], and found that the interaction was still increased when compared to untreated samples or a 10 minute heat shock (Figure S2B, bottom panel). This increase in the Hsf1-Hsp90 interaction after a prolonged heat shock (Figure 2B), accompanies the decline in Hsf1 activity and the down-regulation of the heat shock response [49].

To determine whether other components of the Hsp90 chaperone machinery might also interact with Hsf1, we focused on Hsp70, which operates within the Hsp90 chaperone system [74], and has been implicated in Hsf1 regulation. Indeed, the deletion of *SSA1* and *SSA2,* which encode cytosolic isoforms of Hsp70 derepresses Hsf1 transcriptional activity in *S. cerevisiae* [75]. However, a physical interaction between Hsp70 and Hsf1 has not been reported in any yeast. Therefore, we re-probed our FLAG-Hsf1 immunoprecipitations with an antibody against Hsp70. We found that Hsp70 interacts with Hsf1, but only in response to a prolonged heat shock (Figure 2C, bottom left panel, and Figure S2A). We also re-probed our Hsp90-TAP immunoprecipitations for Hsp70. Our data suggest that Hsp90 and Hsp70 interact in response to prolonged heat shock (Figure 2C, bottom right panel and Figure S2B, top panel).

Given our findings that the Hsf1-Hsp90 interaction strengthens upon heat shock, and that Hsf1 is thought to bind DNA constitutively [44], one might predict that Hsp90 localises to the nucleus upon heat shock. Indeed, a recent study by Lamoth and colleagues shows Hsp90 nuclear localisation upon a 55°C heat shock in *Aspergillus fumigatus* [76]. Therefore we followed the localisation of Hsp90-GFP in *C. albicans* in response to a heat shock (CaLC1855, Table 1). Under steady state conditions, Hsp90-GFP was distributed throughout the cell, with no obvious localisation (Figure 2D), and this was also the case 10 minutes after a 42°C heat shock. However, 60 minutes post-heat shock, nuclear accumulation of Hsp90-GFP was clearly evident (Figure 2D), and Hsp90-GFP remained in the nucleus 120 minutes post heat shock (Figure S2C). Therefore the dynamics of the nuclear accumulation of Hsp90-GFP correlated with the dynamics of the Hsf1-Hsp90 interaction. These data reinforce the view that Hsp90 and the Hsp90 chaperone machine plays an important role in the down-regulation of Hsf1 and the heat shock response.

### How is Hsf1 activated?

**Figure 3:**
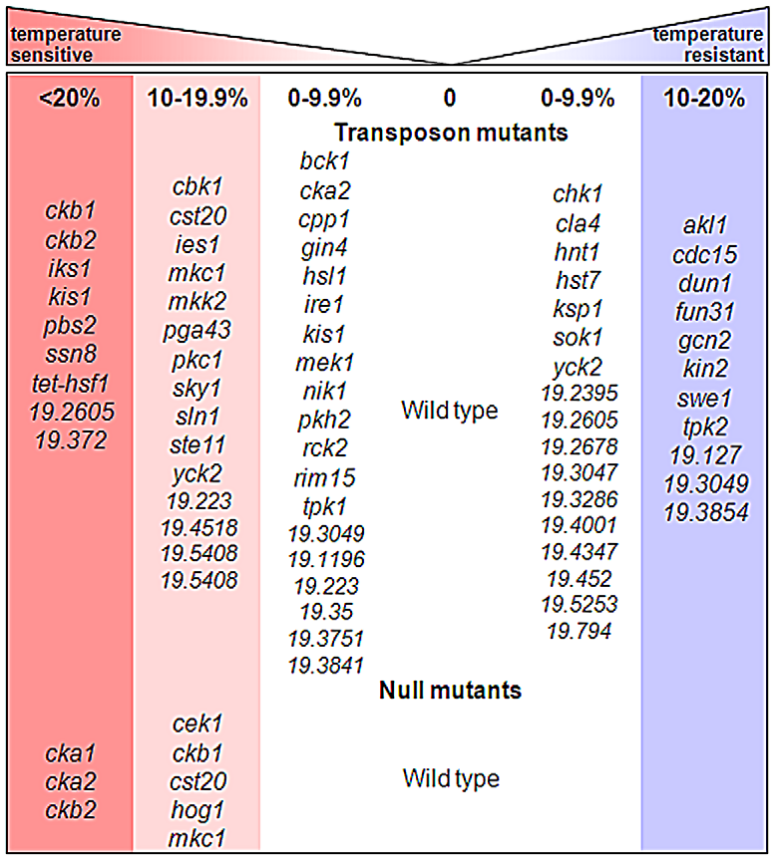
Key MAP kinase signalling pathways contribute to thermal adaptation in *C. albicans*. Figure depicting protein kinase mutants screened for temperature phenotypes. Temperature sensitivity was assayed by monitoring the growth of *C. albicans* mutants following a 30°C–42°C heat shock. Triplicate primary screens examined transposon mutants [57], and triplicate secondary screens tested *C. albicans* null mutants (Table 1). Temperature resistant kinases are displayed in the blue column, kinases with no apparent role in thermal adaptation are displayed in the pale columns, and temperature sensitive kinases are present in the pink (10–19%) to red (>20%) columns.

The above observations indicate that while Hsp90 down-regulates Hsf1, Hsf1 activation is mediated by Hsp90-independent mechanisms. How then is Hsf1 activated? *C. albicans* Hsf1 is activated by phosphorylation [41], but the protein kinase responsible for this in yeast remains unknown [52], [53]. To determine which kinase is responsible for Hsf1 phosphorylation in *C. albicans*, we exploited the recent availability of the *C. albicans* transposon insertion kinase mutant collection which was kindly provided by Aaron Mitchell [58]. This collection of mutants which comprises homozygous insertion or deletion mutations in 67 protein kinase genes and 13 protein kinase-related genes, were screened for temperature sensitivity by monitoring their growth following a one hour 30°C–42°C heat shock. Mutants that consistently displayed a >10% growth defect relative to the wild-type controls in three independent screens were considered temperature sensitive, and those that consistently displayed >10% growth acceleration were considered temperature resistant (Figure 3). The *tetO*-*HSF1* mutant (CLM60-1: Table 1) was always temperature sensitive, and the known temperature sensitivity of *mkc1* mutants [32] was consistently recapitulated. (Mkc1 is the orthologue of *S. cerevisiae* Slt2 [32].) These observations leant weight to the validity of the output from this screen. In this study we focussed on those kinases whose inactivation confers temperature sensitivity.

Numerous kinase mutants displayed significant and reproducible temperature sensitivity in our screen, notably the casein kinase subunits as well as components of key MAP kinase pathways such as the cell wall integrity pathway (Mkc1, Pkc1), the osmotic stress pathway (Pbs2) and the starvation and cell wall stress pathway (Ste11) (Figure 3). Several downstream kinases of these pathways were absent from the mutant collection, prompting us to perform a secondary screen of null mutants. This secondary screen confirmed the temperature sensitivity of all of the transposon mutants tested from the primary screen (Figure 3), including the casein kinase subunits (Cka1/2 and Ckb1/2) and Mkc1. Furthermore, the secondary screen tested the MAP kinases Cek1 and Hog1 (which were missing from the collection of transposon mutants), thereby confirming the involvement of these pathways in *C. albicans* thermotolerance (Figure 3).

Our next aim was to test whether any of the kinases identified in the above screen are responsible for Hsf1 phosphorylation in response to heat shock. Hsf1 was FLAG_3_-tagged at its N-terminus in CAI4 (MLC67) and in each of the following temperature sensitive null mutants: *mkc1* (MLC30), *hog1* (MLC15), *cek1* (MLC21), *cka1* (MLC24), *cka2* (MLC27), *ckb1* (CaLC2259) and *ckb2* (CaLC2261) (Table 1). Each of these mutants was then subjected to a 30°C–42°C heat shock, and Hsf1 phosphorylation assayed at 0, 10, 30 and 60 minutes post-heat shock (Figure S3). All mutants displayed similar Hsf1 phosphorylation dynamics to wild-type cells, indicating that none of the kinases alone is essential for Hsf1 phosphorylation. Therefore, there might be functional redundancy with respect to Hsf1 phosphorylation during heat shock. Alternatively, Hsf1 might be phosphorylated by an essential kinase that was not represented in the kinase mutant collection. These observations also suggest that the MAP kinase pathways might contribute to thermal adaptation in *C. albicans* by mechanisms other than via Hsf1 phosphorylation and the activation of the heat shock regulon.

### MAP kinase pathways display differential activation profiles during thermal adaptation

Next we explored how the signalling pathways contribute to thermotolerance. A recent and elegant study by Diezmann and colleagues [62] demonstrated that casein kinase 2 (CK2) regulates Hsp90 phosphorylation and activity. Therefore, the mutations in CK2 subunits (*cka1*, *cka2*, *ckb1* and *ckb2*) probably reduce thermotolerance by interfering with Hsp90 function. In addition to regulating Hsf1 (Figures 1 and 2), which is critical for thermal adaptation [49], Hsp90 has numerous other client proteins, some of which may contribute to thermotolerance [62].

**Figure 4:**
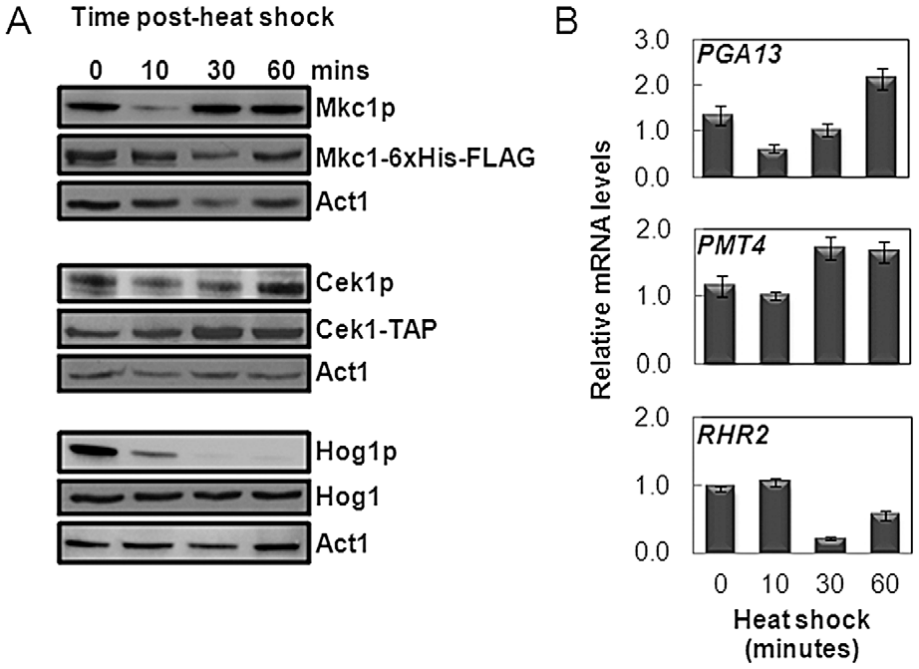
Differential activation profiles of MAP kinases in response to heat shock. (A) Phosphorylation of *C. albicans* Mkc1, Cek1 and Hog1 during a 30°C–42°C heat shock revealed by western analysis using phospho-specific antibodies. Total kinase levels were monitored using antibodies against total Hog1, FLAG-tagged Mkc1 and TAP-tagged Cek1. Actin served as an internal loading control. (B) Expression of target genes for Mkc1 (*PGA13*), Cek1 (*PMT4*) and Hog1 (*RHR2*) during a 30°C–42°C heat shock, as determined by qRT-PCR of the corresponding transcripts relative to the internal *ACT1* mRNA control.

We therefore focussed on the roles of the three MAP kinase pathways that were highlighted by our screen: the cell wall integrity Mkc1 pathway, the osmolarity/oxidative stress Hog1 pathway and the starvation/cell wall response Cek1 pathway. First we monitored the activation of each MAP kinase during heat shock. Wild-type CAI4 cells were subjected to a 30°C–42°C heat shock, proteins extracted at 0, 10, 30 and 60 minutes, and MAP kinase phosphorylation probed by western blotting. Each MAP kinase responded differently to the thermal upshift (Figure 4A). Mkc1 was rapidly dephosphorylated before being re-phosphorylated in the longer term. Cek1 phosphorylation levels remained relatively stable. Hog1 was rapidly dephosphorylated, and was not reactivated during the one hour period examined (Figure 4A). These data were entirely consistent with previous studies that have reported effects of temperature on Hog1, Cek1 and Mkc1 phosphorylation [22], [30], [77], [78]. Significantly, total kinase levels remain unchanged during the 60 minute heat shock.

To further validate the effects of heat shock upon these signalling pathways, we examined the expression of key MAP kinase targets: *PGA13* is an Mkc1 target [78], *PMT4* is a Cek1 target [79] and *RHR2* is a Hog1 target [30]. RNA was extracted from cells 0, 10, 30 and 60 minutes after a 30°C–42°C heat shock, and transcript levels assayed by qRT-PCR relative to the internal *ACT1* mRNA control. The levels of these targets reflected the activation profiles of the corresponding MAP kinase (Figure 4B).

### Cross-talk between the Mkc1, Hog1 and Cek1 pathways during heat shock

**Figure 5:**
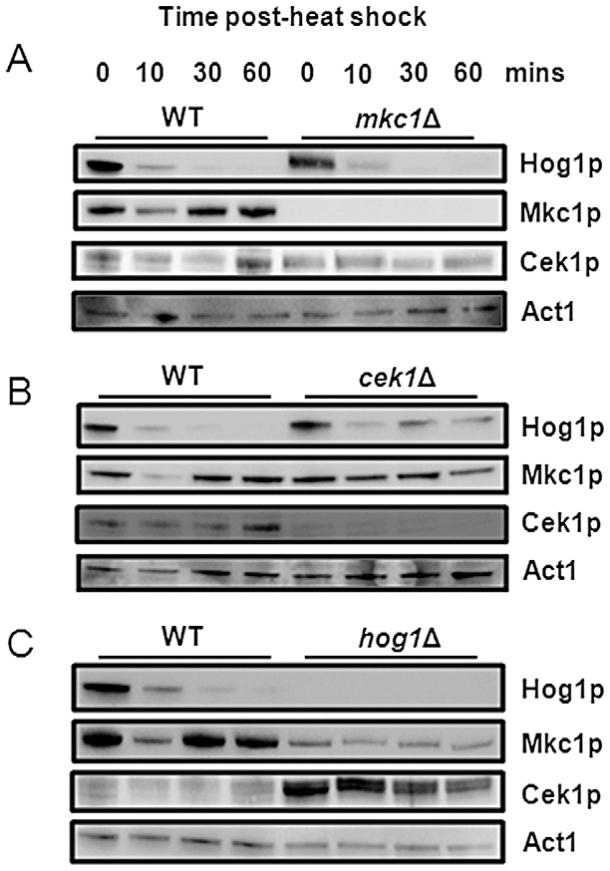
Cross-talk between MAP kinase pathways during heat shock. Differential MAP kinase activation in response to a 30°C–42°C heat shock in MAP kinase mutants. (A) Activation of Hog1, Mkc1 and Cek1 was determined in an *mkc1*Δ mutant relative to the internal Act1 control. (B) Activation of Hog1, Mkc1 and Cek1 was assayed in a *cek1*Δ mutant relative to the internal Act1 control. (C) Activation of Hog1, Mkc1 and Cek1 was determined in a *hog1*Δ mutant relative to the internal Act1 control.

MAP kinase signalling is often represented in terms of linear pathways. However, in *C. albicans*, as in other organisms there is emerging evidence for cross-talk between these pathways [22], [80], [81], [82]. Therefore we tested whether they interact during thermal adaptation by comparing the phosphorylation status of the terminal kinases in wild type and MAP kinase mutants following a 30°C–42°C heat shock (Figure 5). First, we examined Cek1 and Hog1 activation in an *mkc1* mutant (Figure 5A). Mkc1 inactivation did not affect the responses of either Hog1 or Cek1. Second, we monitored Hog1 and Mkc1 phosphorylation in a *cek1* mutant (Figure 5B). After Cek1 inactivation, the transient dephosphorylation of Mkc1 was inhibited. Hog1 phosphorylation levels still declined over the duration of the heat shock. Third, we tested the effects of Hog1 inactivation upon Mkc1 and Cek1 (Figure 5C). Mkc1 phosphorylation was minimal and remained low when *hog1* cells were exposed to the heat shock. In contrast, we observed that Cek1 was hyperphosphorylated in *hog1* cells, as reported previously [22], [81], [83]. Our data reveal that there are significant interactions between the MAP kinase signalling pathways during thermal adaptation.

### Thermal adaptation affects Mkc1, Hog1 and Cek1-mediated resistance to other stresses

**Figure 6:**
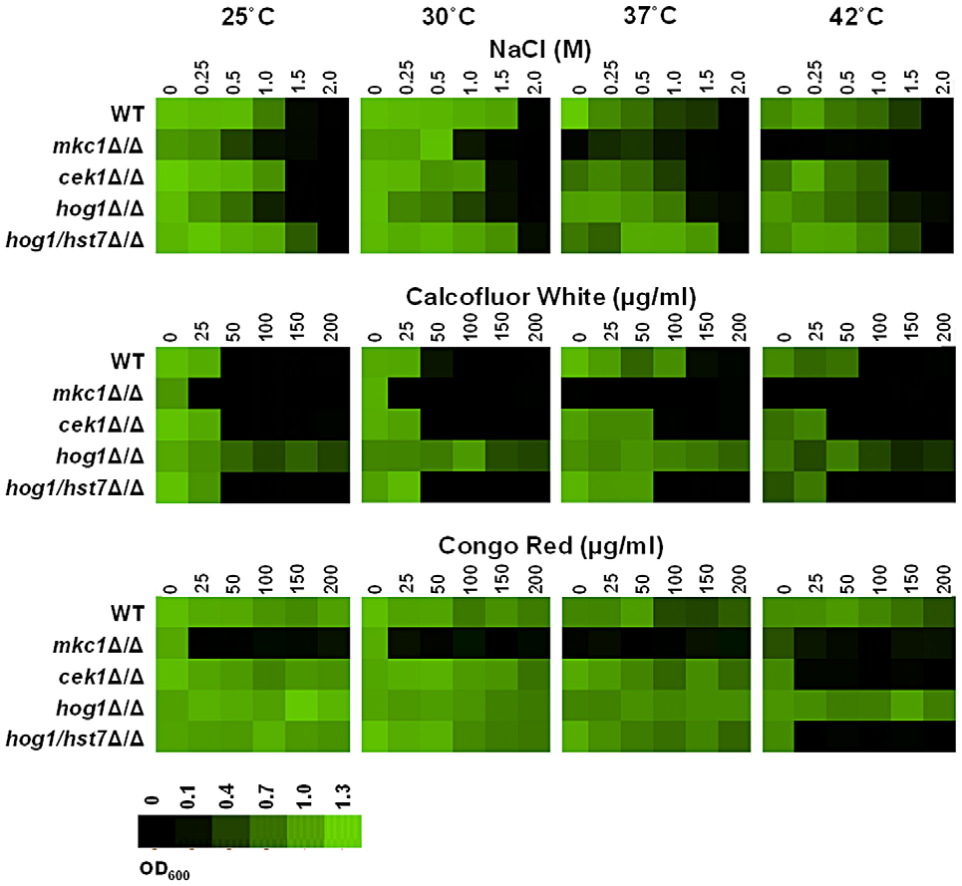
Impact of MAP kinase cross-talk upon stress resistance during thermal adaptation. Cek1 signalling is required for the Calcofluor White resistance of *hog1* cells at high temperatures. MIC assays were performed in YPD medium supplemented with different concentrations of NaCl, Calcofluor White or Congo Red. Plates were incubated statically at 25°C, 30°C, 37°C and 42°C for 48 hours. For each strain, optical densities were averaged for duplicate measurements and growth is quantitatively displayed with colour as indicated with the colour bar. Data are representative of three biological replicates. WT, wild type (NGY152: Table 1).

Given this cross-talk and the modulation of MAP kinase activities after thermal up-shifts, we reasoned that ambient temperature is likely to influence the resistance of *C. albicans* cells to those stresses normally associated with these signalling pathways. For example, Hog1 signalling mediates osmotic stress adaptation [84], and the Mkc1 and Cek1 pathways promote resistance to the cell wall stresses Calcofluor White and Congo Red [22]. Therefore we performed MICs to test the effects of ambient temperature upon the resistance of wild-type (NGY152: Table 1), *hog1, cek1* and *mkc1* cells to these stresses (Figure 6). The *mkc1* cells were temperature sensitive, as reported [32], [84]. Also, as reported previously, *hog1* cells were sensitive to NaCl [84]. Furthermore, *mkc1* and *cek1* cells displayed sensitivity to Calcofluor White [22], [81], [83]. These controls faithfully replicated previous observations.

We observed that ambient temperature significantly influences the sensitivity of *C. albicans* cells to cell wall, but not osmotic stresses (Figure 6). Firstly, wild-type cells were more sensitive to Calcofluor White at lower temperatures (25°C and 30°C). Secondly, *hog1* cells were relatively resistant to Calcofluor White at all temperatures tested, and phenocopied wild type cells on Congo Red. Thirdly, *cek1* cells were resistant to Congo Red at most temperatures, but sensitive at 42°C. Clearly ambient temperature significantly affects cell wall stress resistance. These data reinforce the notion of cross-talk between thermal and cell wall stress signalling pathways.

We reasoned that the Calcofluor resistance of *hog1* cells at low temperatures (Figure 6) might be Cek1 dependent. This is because inactivation of Hog1 led to elevated Cek1 phosphorylation levels (Figure 5C), and Cek1 promotes Calcofluor White resistance (Figure 6). *Hog1* and *cek1* mutations are synthetically lethal [81], and hence we could not examine a *hog1 cek1* double mutant. Therefore, instead we tested the phenotype of a *hog1/hog1 hst7/hst7* double mutant, in which Cek1 signalling is blocked [81]. As predicted, the inactivation of Cek1 signalling attenuated the Calcofluor White resistance of *hog1* cells at low temperatures (Figure 6). This indicates that Hog1 inactivation promotes cell wall stress resistance at low temperatures via Cek1 signalling. These data reinforce the importance of cross-talk between the MAP kinase signalling pathways and highlight the relevance of this cross-talk for thermal adaptation.

### MAP kinase cross-talk affects acquired tolerance to heat shock and other stresses

**Figure 7:**
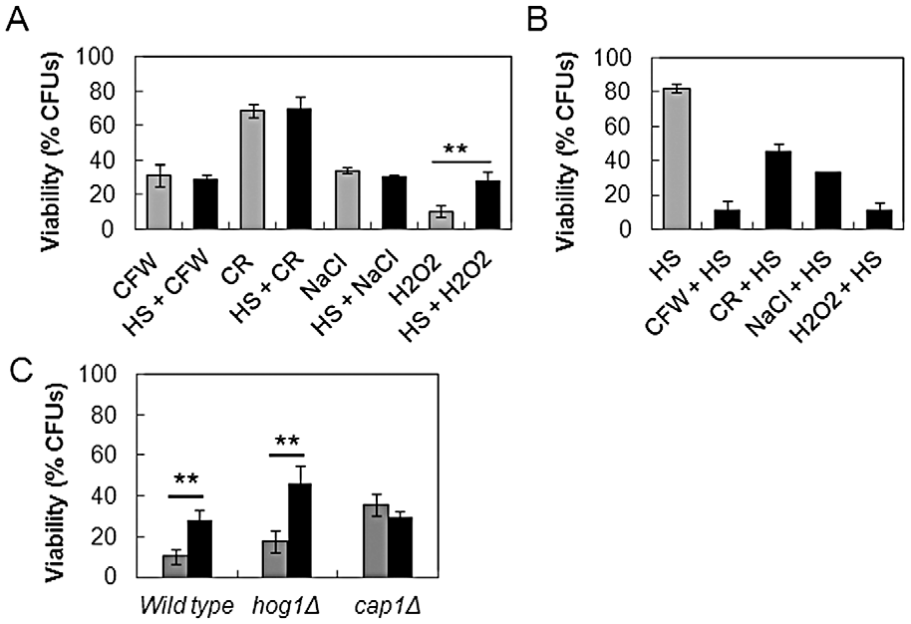
The cross-talk between thermal adaptation and cell wall stress resistance is not mediated via stress cross-protection. (A) Stress cross-protection in *C. albicans* wild-type cells (NGY152: Table 1) was not observed for cells pre-treated with a heat stress and then subjected to cell wall stress, but was observed for cells exposed to a secondary oxidative stress. The data represent cell survival after exposure to a 30°C–42°C heat shock followed by a subsequent cell wall (CFW, CR), osmotic (NaCl) or peroxide (H_2_O_2_) stress (see Materials and Methods) and the data are expressed relative to unstressed cells (dark bars). Control cells (grey bars), were not exposed to the prior 30°C–42°C heat shock. (B) The reciprocal assay was performed, whereby *C. albicans* wild type cells were exposed to a prior stress (cell wall: CFW, CR; osmotic: NaCl; peroxide: H_2_O_2_) followed by a 30°C–42°C heat shock (dark bars). These data are expressed relative to unstressed cells. Control cells (grey bars) correspond to cells exposed only to the 30°C–42°C heat shock. (C) Wild type, *hog1*Δ (JC50) or *cap1*Δ cells (JC128: Table 1) were pre-treated with a 30°C–42°C heat shock, followed by a H_2_O_2_ stress (dark bars). Control cells (grey bars) were not exposed to the 30°C–42°C heat shock. The data represent the level of survival compared to unstressed cells. All data are the means from three independent assays: ** paired, two-tailed t-test, p<0.01.

In some yeasts, the core transcriptional response to stress underpins the phenomenon of stress cross-protection, whereby exposure to one stress protects the cell against subsequent exposure to an alternative type of stress via the up-regulation of key stress response genes [2], [4], [15], [16]. The core stress response is limited in *C. albicans* [19]. Nevertheless, it remained conceivable that the effects of ambient temperature upon the resistance of *C. albicans* to certain stresses might be mediated through stress cross-protection. To test this, mid-exponential *C. albicans* cells (NGY152: Table 1) were subjected to a 30 minute 30°C–42°C heat shock and subsequently exposed to a cell wall stress (Congo Red or Calcofluor White), osmotic stress (NaCl), or oxidative stress (hydrogen peroxide). Cell wall or osmotic stress resistance was not enhanced by prior exposure to heat shock (Figure 7A). Furthermore, prior exposure to cell wall or osmotic stress resistance did not enhance resistance to a subsequent heat shock (Figure 7B). This was consistent with our observation that ambient temperature does not significantly affect osmotic stress resistance (Figure 6), and indicated that the influence of ambient temperature upon Calcofluor White resistance is not mediated by stress cross-protection. This was consistent with the divergent core stress response in *C. albicans* [3], [19].

We included oxidative stress as a control in the above experiments because a prior heat shock has been reported to protect *C. albicans* against peroxide stress [3]. The mechanisms by which a heat shock protects cells against a subsequent oxidative stress have not been elucidated. We noted that two uncharacterised genes that are induced by oxidative stress are also up-regulated by heat shock: orf19.7882 and orf19.7085 [3], [41], [85]. Both genes are induced in response to oxidative stress in a Cap1-dependent fashion, and are down-regulated by Hog1. Therefore we tested whether Hog1 and Cap1 are required for the observed stress cross-protection (Figure 7C). The *hog1* mutant (JC50) displayed a comparable increase in survival to wild type cells when cells were pre-treated with a 30°C–42°C heat shock and then exposed to hydrogen peroxide. In contrast, *cap1* cells (JC128: Table 1) lost this stress cross-protection. Therefore, the acquired resistance to hydrogen peroxide after exposure to heat shock is dependent on Cap1. This probably occurs through the Cap1 dependent up-regulation of oxidative stress genes such as orf19.7882 and orf19.7085 in response to heat shock.

### Cross-talk between thermotolerance and stress adaptation is mediated by Hsp90

What mechanisms are responsible for the cross-talk between thermotolerance and stress adaptation if this is not mediated by stress cross-protection? Hsp90 is a key regulator of thermal adaptation, regulating its own expression via Hsf1 (Figure 1). In addition, Hsp90 modulates the activities of multifarious client proteins [62]. This list of Hsp90 interactors in *C. albicans* includes CK2 subunits [62], Mkc1, a well-defined Hsp90 protein client in *C. albicans* [86], and Hog1 [62]. Mkc1 and Hog1 orthologues are also known Hsp90 client proteins in *S. cerevisiae* [87], [88]. As these protein kinases were identified in our screen as being important for thermotolerance, we reasoned that Hsp90 might play a significant role in coordinating thermal adaptation in *C. albicans*. According to this hypothesis, changes in ambient temperature are expected to influence Hsp90 availability [88] and this in turn modulates the activity of its client proteins [49]. These client proteins include Hog1 and Mkc1 which, when activated, induce expression of target genes that promote cellular adaptation to elevated temperatures and other stresses.

**Figure 8:**
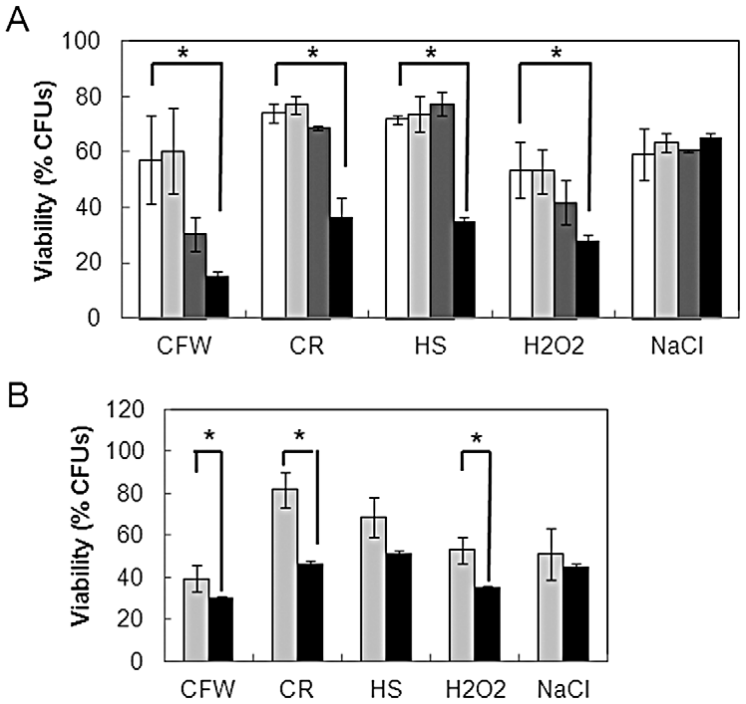
Hsp90 depletion decreases stress resistance. (A) Impact of Hsp90 depletion upon stress resistance (CFUs) was measured following doxycycline treatment of *C. albicans* wild-type and *tetO-HSP90* cells (SN95 and CaLC1411: Table 1): white bars, wild type, no doxycycline; light grey bars, wild type plus 20 µg/ml doxycycline; dark grey bars, *tetO-HSP90* no doxycycline; black bars, *tetO-HSP90* plus 20 µg/ml doxycycline; CFW, 100 µg/ml Calcofluor White; CR, 100 µg/ml Congo Red; HS, 30°C–42°C heat shock; 5 mM H_2_O_2_; 1 M NaCl. (B) Impact of Hsp90 inhibition with the pharmacological inhibitor geldanamycin. Cells were grown for 7 hours in the absence or presence of 10 µM geldanamycin and subjected to the same stresses as in (A). CFUs determined from untreated cells. All data are the means from three independent assays: ** paired, two-tailed t-test, p<0.01.

A clear prediction of this hypothesis is that Hsp90 depletion should attenuate the cell's ability to withstand specific stresses. To test this, *tetO-HSP90* cells were treated with 20 µg/ml doxycycline for 7 hours, by which point Hsp90 levels were significantly reduced (Figure S1A and S1B) and growth was beginning to slow (Figure S1C). These cells were then stressed for one hour with Calcofluor White, Congo Red, a 30°C–42°C heat shock, hydrogen peroxide or NaCl (Materials and Methods). As predicted, Hsp90 depletion significantly attenuated cellular resistance to all of the stresses tested except NaCl when compared to wild type or *tetO-HSP90* cells not treated with doxycycline (Figure 8A).

To test this further, we examined the effects of the Hsp90 inhibitor geldanamycin upon stress resistance. Although the differences were not as dramatic as for genetic depletion of Hsp90 (Figure 8A), similar effects were observed following geldanamycin treatment (Figure 8B). The fact that Hsp90 depletion did not affect osmotic stress resistance was entirely consistent with our previous findings that ambient temperature did not influence osmotic stress resistance (Figure 6), and that there was no stress cross-protection for thermal and osmotic stresses (Figure 7).

**Figure 9:**
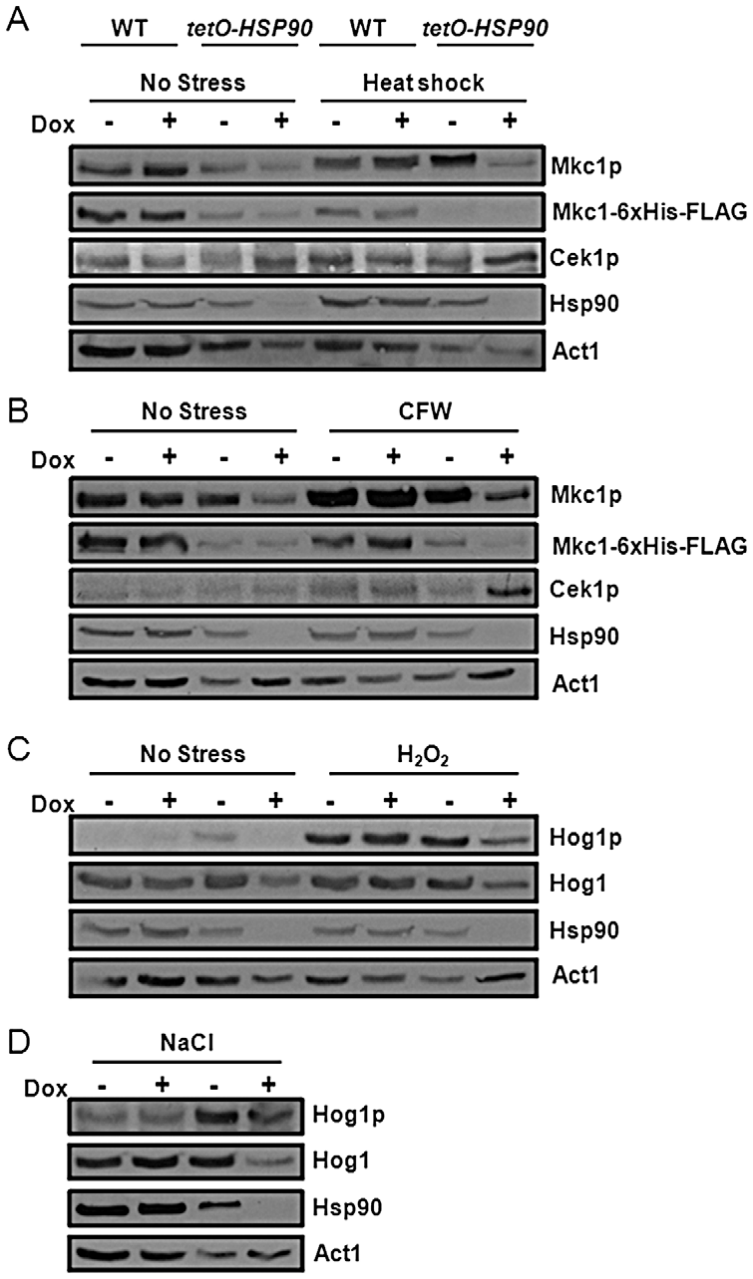
Hsp90 depletion affects MAP kinase signalling in the presence and absence of stress. *C. albicans* wild-type and *tetO-HSP90* cells (SN95 and CaLC1411: Table 1) were treated with 0 or 20 µg/ml doxycycline for seven hours. Mkc1, Cek1 and Hog1 phosphorylation levels were then assayed by western analysis in unstressed cells or cells treated as follows: (A) 30 minute 30°C–42°C heat shock; (B) 30 minutes with 100 µg/ml Calcofluor White (CFW); (C) 10 minutes with 5 mM H_2_O_2_; or (D) 12 minutes with 1 M NaCl. Total Mkc1 levels were assayed using Mkc1-6xHis-FLAG tagged cells in SN95 (CaLC681) and *tetO-HSP90* (caLC648) cells (Table 1). Hsp90 levels and total kinase levels for Hog1 were also examined by western blotting relative to the internal Act1 loading control.

Clearly Hsp90 influences cellular responses to a range of stresses, not only to heat shock. To determine whether these effects are mediated through its client proteins we assessed the impact of Hsp90 depletion upon the activation of known client proteins, Mkc1 and Hog1, and the potential client protein, Cek1 (Figure 9). The basal activation of each MAP kinase was examined in wild type and *tetO-HSP90* (SN95 and CaLC1411: Table 1) in the absence of stress, and following the imposition of stress. Mkc1 activation levels were attenuated following Hsp90 depletion. This was the case in the absence of stress and following heat shock or Calcofluor White treatment (Figures 9A and 9B). This corresponds with a decrease in total Mkc1 kinase levels following Hsp90 depletion. In contrast, Hsp90 depletion had no effect on the levels of Cek1 activation in the absence of stress, but led to an increase in Cek1 phosphorylation following Calcofluor White treatment (Figures 9A and 9B). With regard to Hog1, the basal levels of phosphorylation were maintained following Hsp90 depletion in the absence of stress (Figure 9C), and Hog1 activation was not attenuated in response to osmotic stress although total Hog1 levels decreased (Figure 9D). This was entirely consistent with our other findings, whereby ambient temperature did not affect osmotic tress resistance (Figure 6) and a prior heat shock did not protect cells against a subsequent osmotic stress (Figure 7). However, Hsp90 depletion blocked Hog1 activation following exposure to hydrogen peroxide (Figure 9C). We conclude that Hsp90 depletion exerts differential effects upon Hog1, Mkc1 and Cek1. Furthermore the data are consistent with our prediction that Hsp90 modulates the activities of these MAP kinases.

### Cek1 is an Hsp90 client

**Figure 10:**
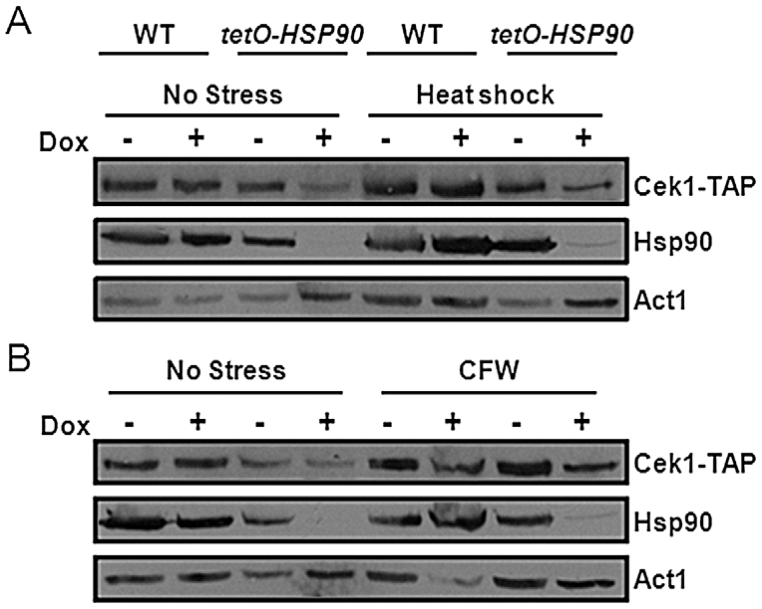
Hsp90 depletion destabilises Cek1. *C. albicans CEK1-TAP* (CaLC2287) and *tetO-HSP90 CEK1-TAP* (CaLC2288: Table 1) cells were treated with 0 or 20 µg/ml doxycycline for seven hours and subjected to western analysis. Decreased Cek1-TAP levels were observed following Hsp90 depletion in unstressed cells and in cells treated as follows: (A) 30 minute 30°C–42°C heat shock; or (B) 30 minutes with 100 µg/ml Calcofluor White (CFW). Hsp90 protein levels were examined confirming significant depletion following doxycycline treatment of *tetO-HSP90* cells. Actin served as the internal loading control.

It was conceivable that Cek1 is an Hsp90 client protein. To test this we determined the impact of Hsp90 depletion on Cek1 stability as Hsp90 client proteins are generally destabilised in the absence of Hsp90 [62], [86], [89]. Cek1 was TAP-tagged at its C-terminus and the specificity of this tagging was verified by western blotting alongside untagged SN95 and CaLC1411 controls (Figure S4). The levels of this Cek1-TAP protein were then monitored in the tet*O-HSP90* strain CaLC1411 (CaLC2288, Table 1) and in wild-type SN95 (CaLC2287, Table 1) cells (Figure 10). Cek1-TAP protein levels decreased in response to Hsp90 depletion in the absence of stress, as well as in response to heat shock and Calcofluor White treatment (Figure 10). This suggested that Cek1 is destabilised by Hsp90 depletion and that Cek1 is a client protein of Hsp90. Therefore the contribution of Cek1 to thermal adaptation appears to be modulated by Hsp90.

### Hsp90 influences cell wall remodelling

**Figure 11:**
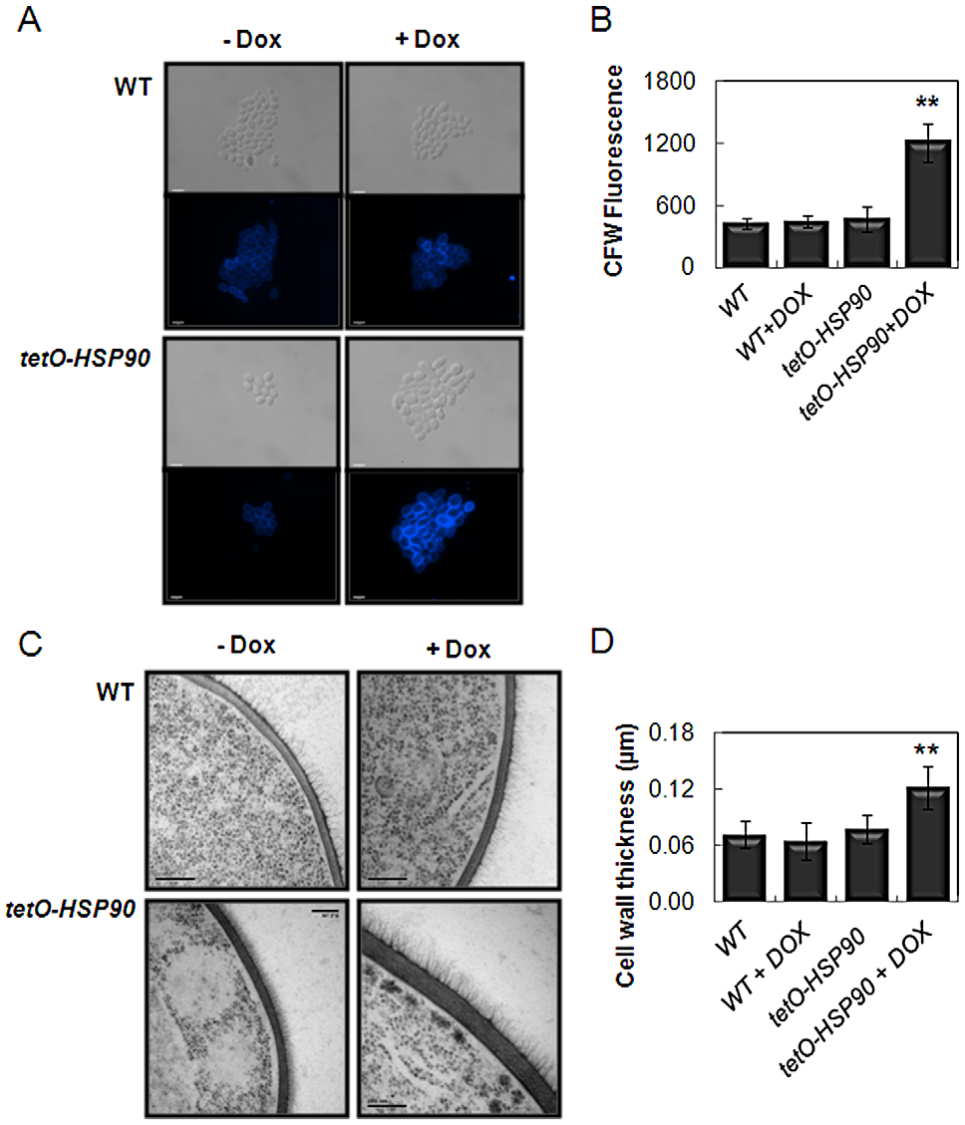
Hsp90 depletion affects cell wall architecture. *C. albicans* wild-type cells (SN95), and *tetO-HSP90* (CaLC1411: Table 1) were treated with 0 or 20 µg/ml doxycycline for seven hours. Chitin levels were assayed by Calcofluor White staining, scale bars are 10 µm (A) and quantification of fluorescence levels (B). Data represent means from fifty cells: ** paired, two-tailed t-test, p<0.01. The architecture of the cell wall was examined by transmission electron microscopy where scale bars are 200 µm (C), and the thickness of the cell wall quantified in n = 30 cells (D): ** paired, two-tailed t-test, p<0.01.

Several observations infer a link between stress adaptation and cell wall architecture in *C. albicans*. For example, the Pkc1/Mkc1 cell wall salvage pathway is activated by certain stresses [58], [66], [90]. Also, the Hog1 stress pathway has been implicated in cell wall biosynthesis [81], partly by regulating chitin synthesis [66], [69]. If Hsp90 depletion modulates Hog1, Mkc1 and Cek1 signalling (Figure 9), and these pathways contribute to cell wall biogenesis, we reasoned that Hsp90 could regulate cell wall architecture. We took two approaches to test this hypothesis. First, we tested the effects of Hsp90 depletion on chitin levels by Calcofluor White staining. Chitin content increased more than two-fold following doxycycline treatment of *tetO*-*HSP90* cells compared to control cells (Figures 11A and 11B). Second, we examined the impact of Hsp90 depletion upon cell wall architecture by transmission electron microscopy (Figure 11C). Cell wall thickness increased two-fold after Hsp90 depletion compared to the controls. Therefore, Hsp90 is essential for normal cell wall structure, providing the first ever link between Hsp90 and cell wall architecture.

## Discussion

Our results illuminate novel functional connections between key cellular regulators required for thermotolerance, and establish distinct roles for Hsp90 in orchestrating short term versus long term mechanisms of thermal adaptation. We provide evidence that Hsf1 is a client protein of Hsp90 for the first time in any fungus, demonstrating that Hsp90 contributes to the short term regulation of thermal adaptation. Furthermore, we identified several key signalling pathways that contribute to thermotolerance in *C. albicans*. These include the Mkc1, Hog1 and Cek1 pathways, each of which plays a role in cell wall integrity. These pathways contribute to thermal adaptation in the longer term via cell wall remodelling, and Hsp90 links many of these pathways, as critical components of these pathways are Hsp90 client proteins. Finally, we establish a role for Hsp90 in cell wall biogenesis for the first time in any organism. Overall, we see that Hsp90 drives short term thermal adaptation via down-regulation of Hsf1, and longer term adaptation through modulation of other client proteins, leading to a more robust cell wall.

Hsf1 is known to activate *HSP90* expression [41] and Hsp90 has been predicted to regulate Hsf1 [49]. Here we demonstrate that inhibiting Hsp90 in *C. albicans*, either pharmacologically or genetically, derepresses Hsf1 (Figure 1), indicating that Hsp90 down-regulates Hsf1. Furthermore, Hsf1 physically interacts with Hsp90 under steady state conditions (Figure 2A), confirming for the first time in any fungus that Hsf1 is an Hsp90 client. What's more, we have shown that this interaction increases during heat shock, and also leads to the recruitment of Hsp70, which binds Hsf1 and Hsp90 during a prolonged heat shock (Figures 2B and S1). This increased interaction correlates with an increase in the levels of both Hsp90 and Hsf1 that occurs during heat shock. Consistent with these findings, we found that Hsp90 accumulates in the nucleus upon a prolonged heat shock (Figure 2C and Figure S1). Similarly, the *S. cerevisiae* Hsp70 chaperone Ssa1 has been shown to enter the nucleus one hour after a 42°C heat shock [91]. This is consistent with our observation that Hsp70 binds Hsf1 and Hsp90 one hour after a 37°C–42°C heat shock. Therefore short-term thermal adaptation involves the activation of Hsf1 by Hsp90-independent mechanisms. Hsf1 induces the expression of Hsp90 and other chaperones that promote protein folding and repair proteotoxic damage [39], [41]. Hsp90 then down-regulates Hsf1 thereby dampening the heat shock response once adaptation is achieved. This regulatory circuit is central to thermal adaptation in *C. albicans* [41], [49] and may be conserved across the eukaryotic kingdom as pharmacological inhibition of Hsp90 derepresses Hsf1 orthologues in *S. cerevisiae* and mammalian systems [52], [55].

**Figure 12:**
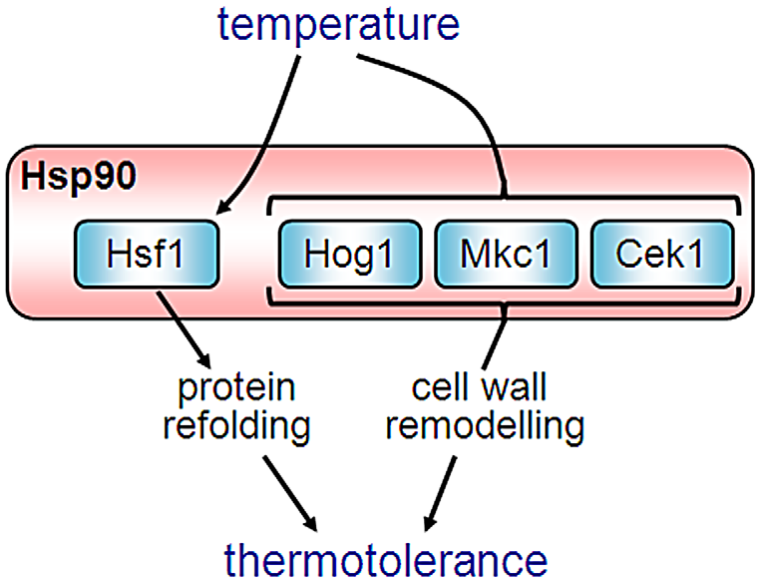
Hsp90 coordinates the activities of multiple signalling pathways that contribute to thermotolerance in *C. albicans* - a model. Hsf1 activation is required for thermotolerance [42]. Hog1, Mkc1 and Cek1 signalling are also required for thermotolerance (Figure 3), but these MAP kinases are not essential for Hsf1 phosphorylation (Figure 4). Instead, these pathways promote thermotolerance in part via cell wall remodelling [81], [90]. Hsp90 coordinates much of this activity. Hsf1 (Figures 1 and 2), Hog1, Mkc1 and Cek1 (Figure 10) are all Hsp90 client proteins [62], [86]. Changes in ambient temperature affect interactions between Hsp90 and Hsf1 (Figure 2), and probably affect Hsp90 interactions with Hog1, Mkc1 and Cek1 [49] thereby modulating the activities of these signalling pathways and their inputs to thermal adaptation. Increases in ambient temperature activate Hsf1, thereby inducing the expression of protein chaperones (HSPs) including Hsp90, which promotes thermal adaptation in the shorter term. Hsp90 then down-regulates Hsf1 and modulates Mkc1, Hog1 and Cek1 signalling, which in the longer term influences cell wall architecture (Figure 11), leading to the thermotolerance of *C. albicans*.

With a view to identifying the kinase responsible for phosphorylating Hsf1 in *C. albicans* we performed a screen for protein kinase mutants that are temperature sensitive (Figure 3). This highlighted several critical regulators on key MAP kinase pathways, including the Mkc1, Hog1 and Cek1 pathways. None of these pathways are essential for Hsf1 phosphorylation (Figure S3), suggesting that there is functional redundancy with respect to Hsf1 activation during heat shock, or that these pathways act independently of Hsf1 in promoting thermotolerance. These pathways are differentially activated during heat shock (Figure 4), and there is cross-talk between these pathways under these conditions (Figure 5). Furthermore, ambient temperature significantly affects the resistance of *C. albicans* cells to cell wall stresses, and these effects are influenced by Cek1, Hog1 and Mkc1 (Figure 6). Each of these MAP kinase pathways is known to contribute to cell wall remodelling [32], [81], [83], [90], and mutations that interfere with cell wall synthesis are known to confer temperature sensitivity upon *C. albicans.* For example, the inactivation of certain protein mannosyltransferases of the PMT family, or the deletion of *OCH1* can confer temperature sensitivity [92], [93]. Additionally, deletion of *SSR1*, a GPI-anchored cell wall protein causes sensitivity to elevated temperatures [94]. These data strongly suggest that Mkc1, Hog1 and Cek1 signalling promotes longer term thermotolerance via the maintenance of a robust cell wall (Figure 12).

As an environmentally contingent hub of protein homeostasis and regulatory circuitry, Hsp90 has profound effects on biology, disease, and evolution. Hsp90 modulates the phenotypic effects of genetic variation in an environmentally responsive manner [95], [96], [97], [98], influencing approximately 20% of observed natural genetic variation and serving to maintain phenotypic robustness and promote diversification [96]. Mkc1 was defined as a protein client of Hsp90 after Mkc1 signalling was shown to contribute to antifungal drug tolerance in *C. albicans* [86]. Hog1 was subsequently shown to be an Hsp90 client protein after Diezmann and co-workers identified this MAPK in a chemogenetic screen of the *C. albicans* Hsp90 interactome [62]. However, Cek1 was not highlighted in this screen. We demonstrate here that, like Mkc1 and Hog1, Cek1 is an Hsp90 client protein (Figure 10). This is in keeping with a recent study by Taipale and colleagues which demonstrates that Hsp90 binds about 60% of mammalian kinases [99]. Therefore we tested whether Mkc1, Hog1 and Cek1 signalling is influenced by Hsp90 during thermal upshifts. We found that Hsp90 does influence the activation of these kinases during heat shock and in response to their respective stresses (Figure 9). Furthermore, Hsp90 depletion influenced the sensitivity of *C. albicans* cells to specific stresses such as cell wall and oxidative stress, as well as to heat shock (Figure 8). Clearly Hsp90 modulates Mkc1, Hog1 and Cek1 signalling and their outputs, and these pathways are known to contribute to cell wall architecture. Therefore, we reasoned that Hsp90 might, in part, influence cell wall remodelling (Figure 12). We confirmed this hypothesis by demonstrating that Hsp90 depletion significantly increases the chitin content and thickness of *C. albicans* cell walls (Figure 11). We note that cell wall robustness does not correlate with cell wall thickness. Indeed Ene and co-workers have recently shown that cell wall architecture is altered by growth on different carbon sources, yielding thinner cell walls that are more robust, leading to increased stress resistance [100]. Therefore, Hsp90 coordinates both short term thermal adaptation (via Hsf1 down-regulation), and long term thermal adaptation (via its client proteins Mkc1, Hog1 and Cek1) (Figure 12).

Additional pathways that were not highlighted in our screen might contribute to thermotolerance. For example some protein kinases that encode essential functions were missing from the transposon library. Indeed, the protein kinase responsible for Hsf1 phosphorylation remains obscure. It is worth noting that as Hsf1 is activated in response to Hsp90 depletion, and as such it is unlikely that the Hsf1 kinase requires stabilisation by Hsp90. One must also note that Hsf1 might be phosphorylated by multiple protein kinases, and hence that there may be functional redundancy with respect to Hsf1 phosphorylation. Indeed, NetPhos2.0 analysis of Hsf1 suggests multiple phosphorylation sites for multiple kinases.

Clearly ambient temperature plays a major role in fungal pathogenicity [42]. Furthermore our data indicate that ambient temperature strongly influences physiological attributes that contribute to fungal pathogenicity, such as a robust cell wall and effective stress adaptation [11], [13], [42], [49]. In addition we show that increases in ambient temperature lead to elevated oxidative stress resistance, but that the reverse is not true (Figure 7). This observation is consistent with the idea of “asymmetric adaptive prediction”, whereby microbes appear to have “learned” over evolutionary timescales that exposure to one stress is likely to be followed by exposure to a second unrelated stress [101]. As a result, exposure to the first stress results in the activation of an adaptive response that prepares the cell for exposure to the second stress [101]. With respect to *C. albicans,* the elevated temperatures associated with localised inflammation, appear to protect the fungal cells against the imminent exposure to oxidative stress that will follow exposure to macrophages and neutrophils.

In conclusion this study reveals new Hsp90 client proteins that play central roles in the control of cellular adaptation: Hsf1 and Cek1. Furthermore, this work provides important new insights into the mechanisms by which Hsp90 coordinates short and long term mechanisms that contribute to thermotolerance in a major fungal pathogen.

## Supporting Information

Figure S1 **Dynamics of Hsp90 depletion in** ***C. albicans tetO-HSP90*** **cells.** (A) Doxycycline-conditional *C. albicans tetO-HSP90* (CaLC1411: Table 1) cells were treated with 20 µg/ml doxycycline, and Hsp90 levels were examined by western blotting and quantified relative to the Act1 internal control. (B) *C. albicans* WT (wild type SN95: Table 1) cells and *tetO-HSP90* cells were treated with 0 or 20 µg/ml doxycycline for 7 hours. Proteins were extracted and probed for Hsp90. (C) Effect of 20 µg/ml doxycycline on the growth of these *C. albicans tetO-HSP90* cells.
(TIF)

Figure S2 **Dynamics of the Hsf1-Hsp90 interaction in** ***C. albicans*** **.** (A) Immunoprecipitation of FLAG-Hsf1 with anti-FLAG M2 affinity agarose during a heat shock shows the interaction of Hsf1 with Hsp70 120 minutes after a 42°C heat shock. (B) FLAG-Hsf1 co-immunoprecipitates with Hsp90-TAP on IgG agarose 0, 10 and 120 minutes post-heat shock. Re-probing these membranes for Hsp70, shows that Hsp70 interacts with Hsp90 120 minutes post-heat shock. (C) Localisation of Hsp90-GFP 120 minutes after heat shock. Cells were treated with a 30°C–42°C heat shock and fixed 120 minutes post-heat shock revealing significant accumulation in the nucleus (localised by DAPI staining). Scale bars, 5 µm.
(TIF)

Figure S3 **Protein kinases that contribute to thermal adaptation in** ***C. albicans*** **are not required for Hsf1 phosphorylation.** Effect of Mkc1, Cek1, Hog1 and CK2 inactivation upon Hsf1 phosphorylation dynamics during a 30°C–42°C heat shock. Wild-type and mutant cells were subjected to a heat shock and proteins harvested at 0, 10, 30 and 60 minutes post-heat shock. All strains exhibited full activation of Hsf1, as seen by the band shift 10 minutes post-heat shock: black arrow, phosphorylated Hsf1; white arrow, non-phosphorylated Hsf1.
(TIF)

Figure S4 **Specific detection of Cek1-TAP.** *C. albicans* wild-type cells (SN95), *CEK1-TAP* (CaLC2287) treated with 0 or 20 µg/ml doxycycline for seven hours (−/+), *tetO-HSP90* (CaLC1411) and *tetO-HSP90 CEK1-TAP* (CaLC2288: Table 1). Western analyses were performed to confirm efficient tagging of Cek1, and that doxycycline does not affect Cek1-TAP protein levels directly. The membrane was reprobed with the internal Act1 control to confirm even loading.
(TIF)

Table S1 **Primers used in this study.**
(PDF)
