# Supplementary figures and images for: Hsp90 Orchestrates Transcriptional Regulation by Hsf1 and Cell Wall Remodelling by MAPK Signalling during Thermal Adaptation in a Pathogenic Yeast
Source: PLoS Pathog. 2012 Dec 27;8(12):e1003069. doi: 10.1371/journal.ppat.1003069 (PMC3531498; doi:10.1371/journal.ppat.1003069)

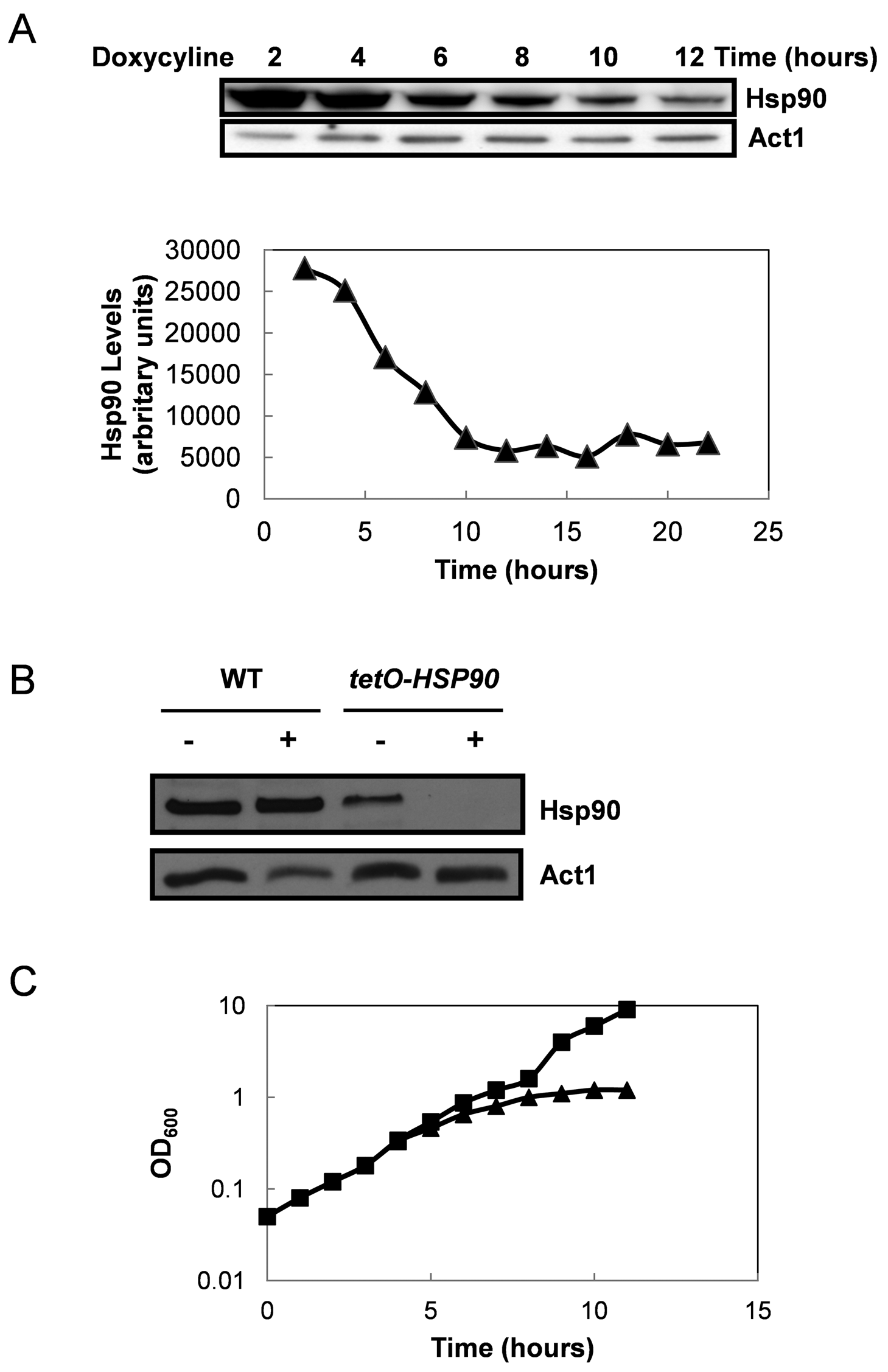

Supplement: Figure S1 — Dynamics of Hsp90 depletion in C. albicans tetO-HSP90 cells. (A) Doxycycline-conditional C. albicans tetO-HSP90 (CaLC1411: Table 1) cells were treated with 20 µg/ml doxycycline, and Hsp90 levels were examined by western blotting and quantified relative to the Act1 internal control. (B) C. albicans WT (wild type SN95: Table 1) cells and tetO-HSP90 cells were treated with 0 or 20 µg/ml doxycycline for 7 hours. Proteins were extracted and probed for Hsp90. (C) Effect of 20 µg/ml doxycycline on the growth of these C. albicans tetO-HSP90 cells. (TIF) [file ppat.1003069.s001.tif]

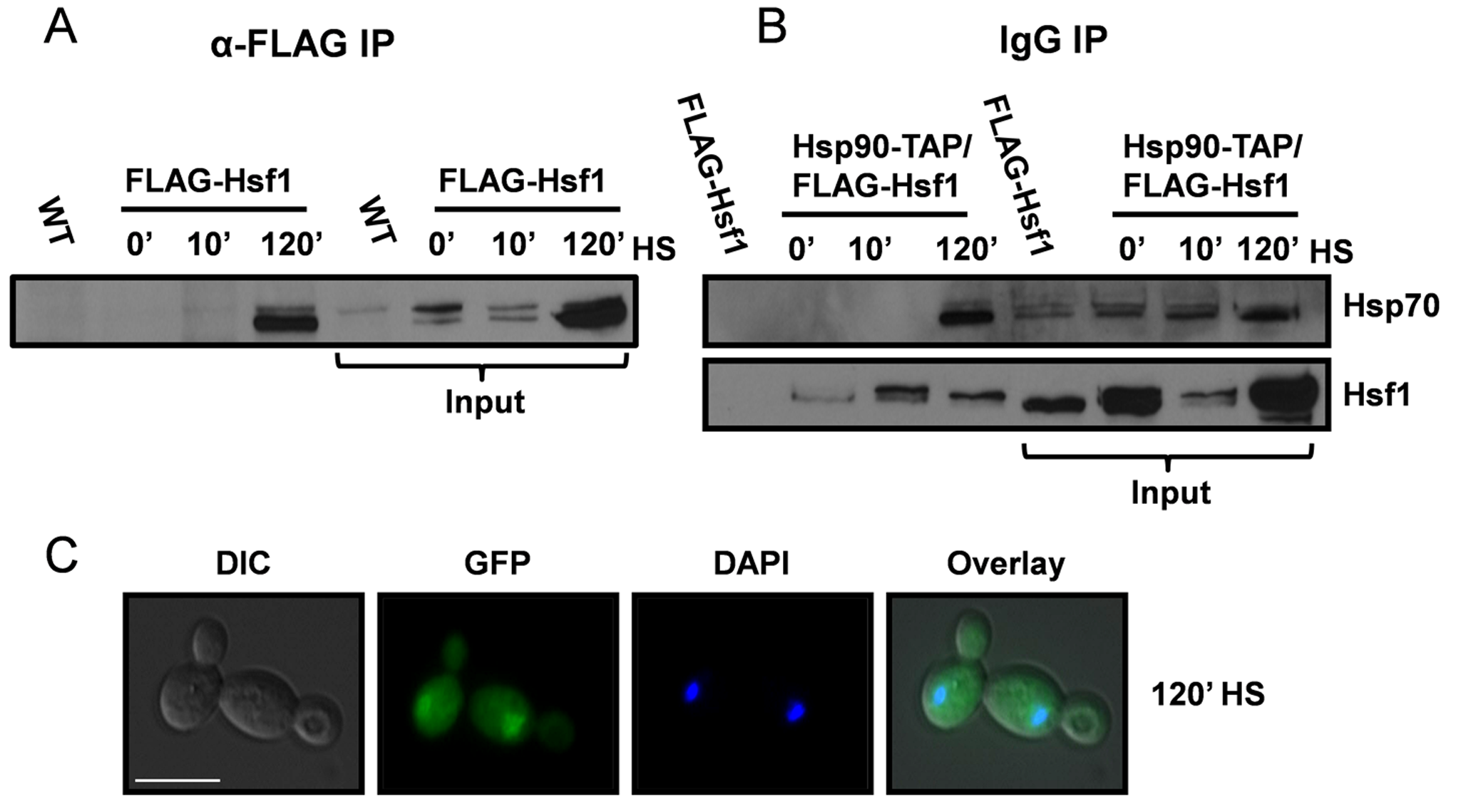

Supplement: Figure S2 — Dynamics of the Hsf1-Hsp90 interaction in C. albicans . (A) Immunoprecipitation of FLAG-Hsf1 with anti-FLAG M2 affinity agarose during a heat shock shows the interaction of Hsf1 with Hsp70 120 minutes after a 42°C heat shock. (B) FLAG-Hsf1 co-immunoprecipitates with Hsp90-TAP on IgG agarose 0, 10 and 120 minutes post-heat shock. Re-probing these membranes for Hsp70, shows that Hsp70 interacts with Hsp90 120 minutes post-heat shock. (C) Localisation of Hsp90-GFP 120 minutes after heat shock. Cells were treated with a 30°C–42°C heat shock and fixed 120 minutes post-heat shock revealing significant accumulation in the nucleus (localised by DAPI staining). Scale bars, 5 µm. (TIF) [file ppat.1003069.s002.tif]

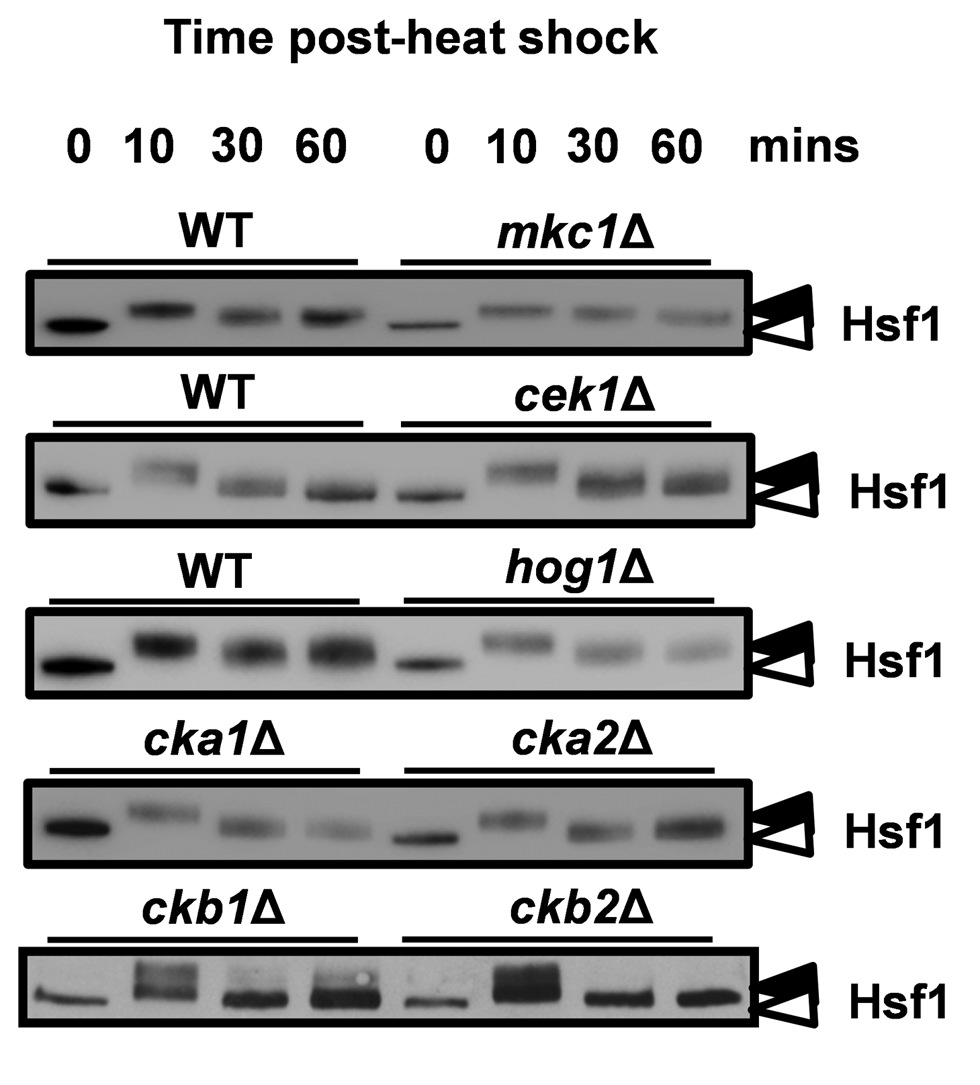

Supplement: Figure S3 — Protein kinases that contribute to thermal adaptation in C. albicans are not required for Hsf1 phosphorylation. Effect of Mkc1, Cek1, Hog1 and CK2 inactivation upon Hsf1 phosphorylation dynamics during a 30°C–42°C heat shock. Wild-type and mutant cells were subjected to a heat shock and proteins harvested at 0, 10, 30 and 60 minutes post-heat shock. All strains exhibited full activation of Hsf1, as seen by the band shift 10 minutes post-heat shock: black arrow, phosphorylated Hsf1; white arrow, non-phosphorylated Hsf1. (TIF) [file ppat.1003069.s003.tif]

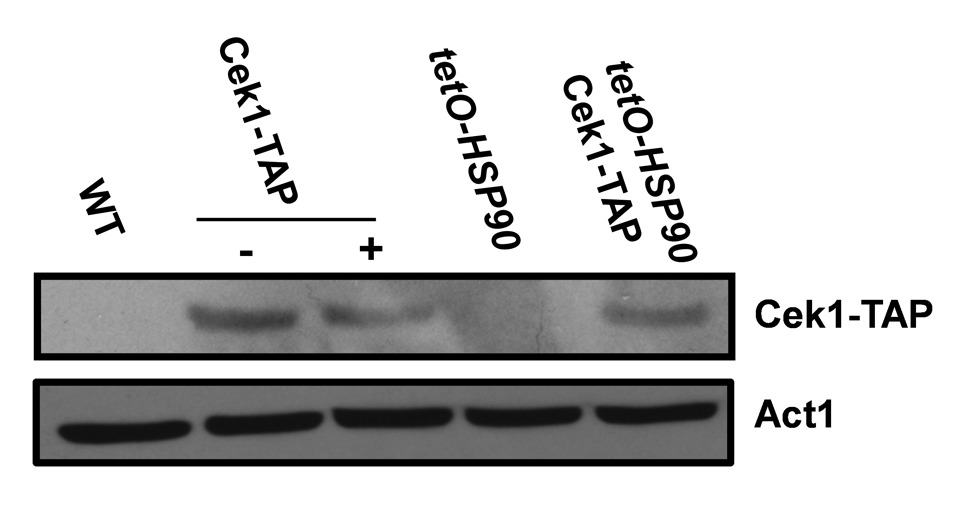

Supplement: Figure S4 — Specific detection of Cek1-TAP. C. albicans wild-type cells (SN95), CEK1-TAP (CaLC2287) treated with 0 or 20 µg/ml doxycycline for seven hours (−/+), tetO-HSP90 (CaLC1411) and tetO-HSP90 CEK1-TAP (CaLC2288: Table 1). Western analyses were performed to confirm efficient tagging of Cek1, and that doxycycline does not affect Cek1-TAP protein levels directly. The membrane was reprobed with the internal Act1 control to confirm even loading. (TIF) [file ppat.1003069.s004.tif]
