# Supplementary material for: Hsp90 Orchestrates Transcriptional Regulation by Hsf1 and Cell Wall Remodelling by MAPK Signalling during Thermal Adaptation in a Pathogenic Yeast
Source: PLoS Pathog. 2012 Dec 27;8(12):e1003069. doi: 10.1371/journal.ppat.1003069 (PMC3531498; doi:10.1371/journal.ppat.1003069)
Supplement: Table S1 — Primers used in this study. (PDF) [file ppat.1003069.s005.pdf]

Table S1 - Primers

oLC1117	CaACT1-1012-F-SacI	CCCGAGCTCGCTATTAAGATCACCAGCCT
oLC1628	Hsf1+114-R	CCAAACAAATCGAGTAATGG
oLC1616	CaHsp90-Linker-GFP-F	GCTACTACTACTGCCTCAACTGACGAACCAGCTGGAGAATCTGCTATGGAAGAAGTTGATGGTGGTGGTTCTAAAGGTGAAGAATTAT
oLC1617	CaHsp90-GFP/NAT-R	TTATGTTATTACTCTCTAGATACACGATATTACAAAACTTATTTAACTAGAAAACTGTAGGTAAAACGACGGCCAGTGAATTC
oLC600	JB-GFP+344-R	CCTTCAAACTTGACTTCAGC
oLC756	HSP90+1051-F	GCTGAAGAGTTGATTCCAGAAT
oLC2285	CaACT1+855-F	GACCTTGAGATACCCAATTG
oLC2286	CaACT1+1076-R	CAGCTTGAATGGAAACGTAG
oLC2256	CaPGA13+468-F	CCAACCACTACATCTACTTC
oLC2257	CaPGA13+665-R	GGAACAACAACTGTAGTTGG
oLC2262	CaPMT4+1151-F	GGAAATAGTTCCAACTCTGG
oLC2263	CaPMT4+1338-R	CTCATTGTAACGTTGTTGGG
oLC2266	CaRHR2+213-F	GAAGAACAAGTCACTGCATG
oLC2267	CaRHR2+399-R	CAACCATTTGGTGGCTAATG
oLC754	HSP90+832-F	CCATCTGATATCACTCAAGATG
oLC755	HSP90+1040-R	AGTGATAAACACTCTACGGACG
oLC1620	Hsp104+1402-F	CGAGCTAGTCATGAACAATTG
oLC1621	Hsp104+1660-R	GGATACCAGTCAATCTAGCA
oLC2292	CaCEK1-TAP-ARG4-F	TAAAATGAAAGATCAATTAACAATTGAAGATTTGAAAAAATTGTTATATGAAGAGATTATGAAGCCATTAGGTCGACGGATCCCCGGGTT
oLC2251	CaCEK1-HA-ARG4-R	ACCAAGCCCAACCTATAGTTTTTAGTTTAGTTTAGTTTAGTTTAGTTTAGCTTAACTTAGCTTGACCTCTTCGATGAATTCGAGCTCGTT
oLC2252	CaCEK1+1143-F	CCTAATGATGAACCAATTAG
oLC2253	CaCEK1+1376-R	CCTATACAACAACAATTATGC
oLC1593	TAP-R	TAAACTTTGGATGAAGGCG
oLC1594	ARG4-F	ATGTTGGCTACTGATTTAGCTG
oLC2364	CaCEK1d Ext F (-436 bp)	CTAGTTCAATTCCCATCAAC
oLC2365	CaCEK1d Ext R (+1326 bp)	CTATTCTTCACACACGTTTAC
oLC274	pJK863down-F	CTGTCAAGGAGGGTATTCTGG
oLC275	pJK863up-R	AAAGTCAAAGTTCCAAGGGG
